# Sodium-Intercalated Vanadium Oxide Coated on Carbon Cloth for Electrode Materials in High-Performance Aqueous Zinc-Ion Batteries

**DOI:** 10.3390/molecules30092074

**Published:** 2025-05-07

**Authors:** Chen Chen, Baoxuan Hou, Ting Cheng, Fei Wu, Yulin Hu, Youzhi Dai, Xiao Zhang, Yuan Tian, Xin Zhao, Lei Wang

**Affiliations:** 1 School of Environmental and Chemical Engineering, Jiangsu University of Science and Technology, Zhenjiang 212100, China; hbx.1999@outlook.com (B.H.); wnchengting@sina.com (T.C.); wufei1224wf@hotmail.com (F.W.); ttyy1974.ok@163.com (Y.T.); zx.just@outlook.com (X.Z.); 18761889001@163.com (L.W.); 2 School of Environmental Ecology, The City Vocational College of Jiangsu, Nanjing 210017, China; 3 College of Chemistry and Chemical Engineering, Anshun University, Anshun 561000, China; huyulin1982@163.com; 4 College of Environment and Resource, Xiangtan University, Xiangtan 411105, China; daiyouzhi202@163.com; 5 Nanjing University and Yancheng Academy of Environmental Technology and Engineering, Yancheng 224000, China; zhangxiao7376@sina.com

**Keywords:** vanadium oxide, aqueous battery, zinc-ion battery, nanowire, intercalated

## Abstract

In this work, novel sodium-intercalated vanadium oxide nanowire electrode materials (NaXV@CC) were successfully designed as cathode materials for Aqueous Zinc-Ion Batteries (AZIBs) through a two-step electrochemical process. The optimized electrode material, Na30V@CC, exhibited superior capacity, excellent rate capability, and outstanding stability. The intercalation of sodium ions into the nanowire lattice induced a significant transformation in the overall nanostructure, leading to altered nanowire morphology. This unique structural design provided abundant active sites and efficient ion transport pathways, thereby enhancing the overall electrochemical performance. The charging and discharging capacities were 343.3 and 330.4 mAh·g^−1^ at 0.2 A·g^−1^, respectively, and the capacity was maintained at 90 mAh·g^−1^ at 8 A·g^−1^. The battery demonstrated exceptional capacity retention over 3000 cycles at 5 A·g^−1^, highlighting its long-term electrochemical stability. Moreover, the overall battery reaction was governed by a combination of diffusion and surface processes. The Na30V@CC battery system demonstrated reduced reaction impedance and improved zinc ion diffusion rates. This study offers valuable insights into enhancing the electrochemical performance of vanadium-based cathodes in AZIBs.

## 1. Introduction

With the rapid proliferation of functional electronic devices, including mobile phones, cameras, and laptops, coupled with the accelerated growth of the electric vehicle industry, substantial attention has been devoted to the development of energy storage devices with high energy density, long-term cycling performance, and enhanced safety [1,2]. Among the various energy storage technologies, secondary rechargeable batteries have gained significant prominence, with lithium-ion batteries (LIBs) being the most extensively developed and widely deployed in electric vehicles and electronic devices [3,4]. Lithium-ion batteries exhibit several key advantages, including high energy density, a broad operating voltage range, and superior cycling stability [5]. However, LIBs also present significant drawbacks. Firstly, lithium is a scarce mineral resource with an uneven global distribution, leading to high extraction costs and significant price volatility. The growing demand for lithium has intensified concerns regarding resource depletion and the long-term sustainability of its supply chain [6,7]. Secondly, the high chemical reactivity of metallic lithium, coupled with the use of flammable organic electrolytes, poses significant safety concerns, including the potential for combustion and explosion. Such incidents have, in some cases, led to severe injuries and fatalities [8,9]. In light of these challenges, the sustainable advancement of the energy storage industry requires the exploration of alternative rechargeable battery technologies that achieve an optimal balance among capacity, cost-effectiveness, safety, and long-term reliability. Aqueous zinc-ion batteries (ZIBs) have emerged as a promising alternative, garnering considerable attention for their potential to address the aforementioned challenges [10,11].

In contrast to lithium-ion batteries that employ flammable organic electrolytes, aqueous batteries utilize metal ion solutions as electrolytes, thereby substantially enhancing safety by reducing the risk of flammability [12,13]. Among various aqueous rechargeable battery systems, ZIBs have attracted considerable research attention due to their high theoretical capacity (820 mAh·g^−1^), suitable redox potential (−0.76 V vs. the standard hydrogen electrode, SHE), abundance of zinc resources, simplified assembly procedure, low environmental toxicity, and high safety performance [14,15]. In aqueous zinc-ion batteries, the electrochemical performance of the cathode material is a critical factor influencing overall battery efficiency, cycling stability, and energy storage capacity [16]. During the charging process, the cathode material must efficiently facilitate reduction reactions and promote the intercalation of zinc ions, while during discharge, it must effectively support the corresponding oxidation reactions to ensure optimal electrochemical performance. The reversible redox process, encompassing the intercalation and deintercalation of zinc ions, is essential to the battery’s capacity and long-term cycling stability [17].

Although AZIBs demonstrate significant potential, their practical performance is often constrained by limitations associated with the cathode material, including inadequate structural stability, low electrical conductivity, and a restricted cycling lifespan. Hence, exploiting suitable cathode materials that fulfill the requirements of high capacity, robust stability, and outstanding rate performance is essential for the effective application of AZIBs. Presently, the cathode materials commonly used in aqueous zinc-ion batteries include Prussian blue analogs, manganese oxides, vanadium oxides, and various organic electrode materials [18,19]. Vanadium oxides and their derivatives offer advantages such as diverse crystal structure, cost-effectiveness, abundant resources, and extensive material variety, making them highly promising cathode candidates for ZIBs [20,21]. These materials facilitate the development of multidimensional structures, enabling efficient intercalation and deintercalation of zinc ions within the V–O structural framework during charge–discharge cycles. Simultaneously, the vanadium element undergoes valence state transitions between +3, +4, and +5, thereby facilitating the redox reactions essential for energy storage [22]. For instance, Guo et al. developed a new cathode material, VO(OH)_2_, that showed superior specific capacity and remarkable long-term stability in aqueous zinc-ion battery applications [23]. Similarly, hydrated calcium vanadate (CaV_6_O_16_·3H_2_O) was prepared by Liang et al. as a cathode material for aqueous zinc-ion batteries, showing remarkable specific capacity and enhanced cycle stability [24]. Moreover, akin to other functional materials, the morphology of active materials is a crucial factor in determining the performance of aqueous zinc-ion batteries. For example, Ma et al. developed a VO_2_ material featuring a distinct flower-like morphology. This unique structure effectively minimized ion transport distances and facilitated enhanced electrolyte infiltration, leading to improved specific capacity and stability of the battery [25]. In addition, Luo et al. synthesized a V_2_O_3_ material with a hierarchical micro-cuboid structure. When employed as a cathode material, this structure exhibited significantly enhanced specific capacity and improved cycling stability in comparison to commercially available V_2_O_3_ [26].

Nevertheless, studies indicate that vanadium oxides still suffer from drawbacks such as low reversible specific capacity, structural instability, and sluggish zinc-ion transport, which continue to impede their performance enhancement in ZIBs [27]. These issues primarily stem from the collapse of the layered crystal structure of the electrode material, induced by the insertion and extraction of zinc ions. Moreover, the strong electrostatic repulsion and significant hydration of Zn^2+^ ions during the discharge/charge process result in sluggish electrochemical kinetics. Metal ion pre-intercalation is a crucial strategy to overcome this challenge [28]. The pre-intercalation of metal ions, including lithium, potassium, sodium, and magnesium, has been identified as a promising approach to enhance the capacity and stability of vanadium-based materials in battery applications. Given that the electrochemical performance of layered vanadium-based materials is significantly influenced by interlayer spacing and intrinsic conductivity, the enhancement of battery capacity, stability, and rate capability can be achieved through the pre-intercalation of metal ions. This improvement is primarily attributed to lattice expansion, crystal structure modification, and morphological transformations [29]. Bai et al. utilized a bimetallic pre-intercalation approach involving Na^+^ and Mg^2+^ ions to synthesize a Na_0.13_Mg_0.02_V_2_O_5_·0.98H_2_O material with an interlayer spacing of 11.67 Å, resulting in a substantial improvement in both the specific capacity and stability of the battery. Acting as interlayer pillars, pre-intercalated ions, and structural water contributed to maintaining the integrity of the cathode material throughout the cycling process [30]. As reported by Ma et al., embedding trace sodium ions into the vanadium oxide layers resulted in increased interlayer spacing, contributing to a significant improvement in specific capacity and prolonged stability of the battery [31]. However, the underlying mechanism by which metal ion pre-intercalation enhances the zinc storage performance of vanadium-based materials remains incompletely understood. In addition, there is a continuous demand for the development of advanced electrode materials that meet the requirements of high capacity, durability, and rapid charge–discharge capabilities in battery technologies. Motivated by the previous research, in this work, a series of novel sodium-intercalated vanadium oxide electrode materials with distinctive nanostructures were successfully anchored onto the surface of carbon cloth fibers and denoted as NaXV@CC. These materials were designed as cathodes for AZIBs using a combined approach of electrodeposition followed by sodium intercalation. A comprehensive analysis of the synthesized electrode materials was performed using advanced characterization techniques to investigate their key attributes, including crystallographic phases, morphological structures and transformations, surface elemental composition, and valence states. Subsequently, the designed materials were systematically evaluated for battery performance, with a focus on charge–discharge capacity, rate capability, and cycling stability. Furthermore, the dynamic behaviors were thoroughly analyzed to gain a deeper insight into the reaction mechanisms and kinetic processes of sodium-intercalated vanadium oxide within the entire battery system. A comprehensive approach, incorporating both experimental and theoretical investigations, revealed that sodium ion intercalation substantially improved the electrochemical performance of vanadium-based materials for AZIBs. This work presents a new approach for optimizing vanadium-based cathode materials in battery storage applications.

## 2. Results and Discussions

**Figure 1:**
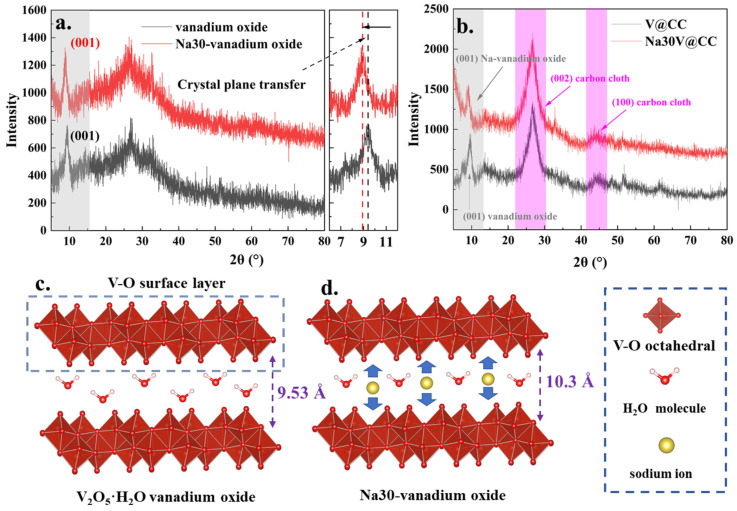
The XRD patterns of powder vanadium oxide and Na30-vanadium oxide (**a**); sheet V@CC and Na30V@CC (**d**); the structural model of vanadium oxide ((**b**): as proved in Figure S2) and Na-vanadium oxide ((**c**): schematic diagram of expected intercalation structure).

Figure 1 displays the XRD analysis results for vanadium oxide and Na30-vanadium oxide in powder form, as well as for the sheet-like materials V@CC and Na30V@CC. As presented in Figure 1a, the XRD patterns of the vanadium oxide synthesized via electrodeposition closely matched the theoretical XRD calculation results for the V_2_O_5_·H_2_O structure (ISCD: 94905, Figure S1). The most prominent diffraction peak, located at approximately 2θ = 9.43°, corresponded to the (001) crystal plane, which represented the surface layer formed by vanadium oxide octahedra. Between these layers, H_2_O molecules were intercalated, resulting in an interplanar spacing of 9.53 Å (Figure 1c).

Following the 30 min electrochemical intercalation process, the XRD pattern of Na30-vanadium oxide closely resembled that of pure vanadium oxide. The primary distinction was a significant shift in the prominent diffraction peak associated with the (001) crystal plane, moving to approximately 2θ = 8.87°. The observed peak shift corresponded to an expansion of the (001) interplanar spacing to 10.3 Å, which could be attributed to the intercalation of sodium ions between the vanadium oxide layers (Figure 1d). This structural modification was anticipated to markedly enhance the capacity of aqueous zinc-ion batteries by facilitating improved ion transport and structural stability [32]. Moreover, Figure 1b presents the XRD patterns of the sheet electrode materials, V@CC and Na30V@CC. The XRD analysis of these materials revealed diffraction peaks corresponding to the (001) crystal planes of vanadium oxide and Na30-vanadium oxide, alongside characteristic peaks of the carbon cloth substrate, specifically the (002) peak at 26.5° and the (100) peak at 44.2°. In addition, other sodium-intercalated electrode materials exhibited similar behavior (see Figure S3). It was evident from these findings that V@CC, Na30V@CC, and other related materials were composites consisting of vanadium oxide and carbon cloth, synthesized by the processes of electrodeposition followed by sodium ion intercalation.

**Figure 2:**
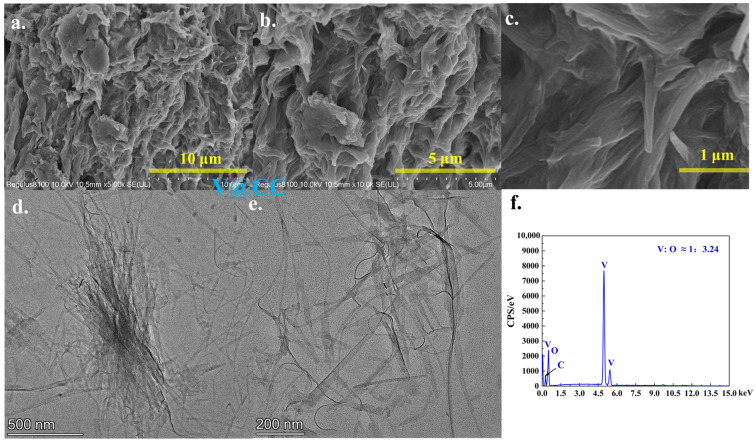
The SEM (**a**–**c**) and TEM (**d**,**e**) morphology analysis results of V@CC; SEM-EDX element area analysis results of V@CC ((**f**): area of (**b**)).

Figure 2 and Figure S4 present the SEM and TEM morphological characterization results of the V@CC material. As observed, after the initial electrodeposition, the V@CC material exhibited an irregular, amorphous microstructure that adhered to the surface of the carbon cloth fibers (Figure S4). The elemental composition determined by EDX (Figure 2f) confirmed the presence of vanadium and oxygen with an approximate atomic ratio of V:O ≈ 1:3.24, uniformly distributed across the entire area (Figure S5). In addition, further insights from TEM analysis (Figure 2d,e) elucidated that the irregular structure of V@CC material consisted of densely packed and aggregated nanowires, approximately 20 nm in diameter and 1–2 μm in length. Although these nanowires exhibited a high specific surface area, their pronounced aggregation and irregular stacking substantially diminished the available effective reaction surface area. These findings unequivocally confirmed the successful synthesis of vanadium oxide compounds with stacked nanowire morphology on the carbon cloth substrate via the electrodeposition process.

**Figure 3:**
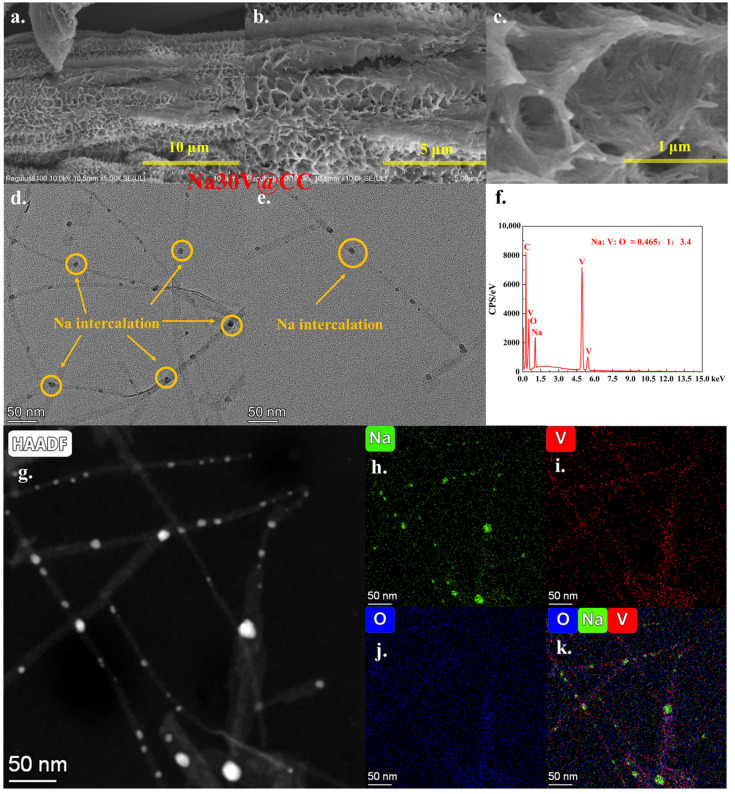
The SEM (**a**–**c**) and TEM (**d**,**e**) morphology analysis results of Na30V@CC; SEM-EDX element area analysis results of Na30V@CC ((**f**): area of (**b**)), and TEM-EDX analysis area (**g**) and element mapping distribution results (**h**–**k**) of Na30V@CC.

Following the second electrochemical intercalation step, the Na30V@CC material, in contrast to conventional vanadium-based cathode materials [28,29,31], exhibited pronounced morphological transformations relative to V@CC. Notably, the surface developed numerous wrinkles approximately 200 nm in thickness, which contributed significantly to the surface area and provided abundant active sites for battery reactions (Figure 3a–c). Moreover, the results of the SEM-EDX area analysis (Figure 3f and Figure S6) demonstrated a uniform spatial distribution of sodium, vanadium, and oxygen across the examined region, with an atomic ratio of Na:V:O at approximately 0.465:1:3.4. This ratio closely aligns with the theoretical molecular composition of Na_0.93_V_2_O_5_·1.8H_2_O, thereby providing preliminary evidence for the successful intercalation of sodium ions into the vanadium oxide crystal lattice. Further structural and elemental information was acquired through TEM analysis. As demonstrated in Figure 3d,e, following the second sodium intercalation, the nanowire structure was retained; however, two significant alterations were noted: first, there was a marked reduction in the degree of nanowire aggregation, and second, multiple dark spots emerged along the central region of the nanowires. The initial observation could be attributed to the notable morphological changes identified in Na30V@CC. As the aggregation of nanowires diminished, an increased active surface area was exposed, leading to the formation of nano-wrinkles (Figure 3a–c). The second observation provided compelling evidence of sodium intercalation. Elemental mapping from TEM-EDX analysis (Figure 3g–k) confirmed that within the region illustrated in Figure 3g, both vanadium (Figure 3i) and oxygen (Figure 3j) were homogeneously dispersed across the nanowire architecture. Sodium was also detected throughout the nanowires, though its intensity was notably elevated at specific particles (Figure 3h). The multi-element analysis presented in Figure 3k further substantiated this finding. The results unequivocally demonstrated that sodium ions were effectively intercalated into the vanadium oxide nanowires during the second electrochemical step. Moreover, it could be inferred that sodium intercalation served as the principal factor contributing to the observed decrease in nanowire aggregation.

Furthermore, the morphology of products following the electric intercalation process exhibited time-dependent evolution. As illustrated in Figure S7, after 20 min of reaction, the sodium intercalation process initiated the formation of a layered morphology, though this effect was localized and did not result in the formation of continuous wrinkles. In contrast, extending the reaction time to 40 min resulted in Na40V@CC electrode material displaying considerably elongated and continuous wrinkled structures, which were notably distinct from those observed in Na20V@CC and Na30V@CC electrode materials.

Simultaneously, the EDX elemental composition results (Figure 3f, Figures S8 and S9) indicated that the sodium content increased with prolonged intercalation time, as evidenced by the atomic ratios of Na:V:O, approximately 0.36:1:3.34 for Na20V@CC and 0.57:1:3.1 for Na40V@CC. These findings suggested that the observed morphological changes might be closely associated with varying degrees of sodium intercalation within the nanowires, which were modulated by the duration of the intercalation process. Moreover, SEM-EDX elemental mapping analysis of Na20V@CC (Figure S10) and Na40V@CC (Figure S11) confirmed the presence of sodium, vanadium, and oxygen in the synthesized electrode materials, with a uniform spatial distribution across the analyzed region. Based on an integrated analysis of SEM and TEM, it could be concluded that the initial electrodeposition step was efficacious in depositing irregular vanadium oxide, composed of stacked nanowire structures, onto the surface of carbon cloth. Subsequently, in the second electrochemical reaction step, sodium ions were effectively intercalated into the nanowire lattice, inducing significant structural modifications that led to a pronounced transformation in the overall nanomorphology.

**Figure 4:**
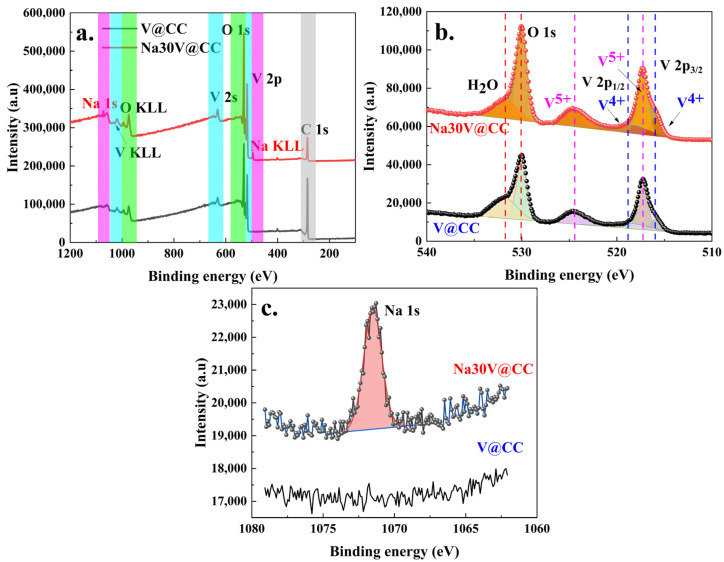
The XPS full spectrum scanning of V@CC and Na30V@CC materials (**a**); high-resolution XPS results of O 1s (**b**), V2p (**b**), and Na 1s (**c**) of V@CC and Na30V@CC materials.

The elemental composition and valence states on the surface of the materials were characterized through X-ray photoelectron spectroscopy (XPS). The XPS analysis results for V@CC and Na30V@CC electrode materials were displayed in Figure 4. The high-resolution full-spectrum scans (Figure 4a) revealed prominent peaks corresponding to vanadium (V 2p and V 2s), oxygen (O 1s), and carbon (C 1s) in both V@CC and Na30V@CC spectra. The sodium (Na 1s) peak was exclusively detected in Na30V@CC as well as in other sodium-intercalated electrode materials (Figure S12). In addition, the carbon signal was ascribed to the conductive carbon cloth substrate, whereas the vanadium-oxygen compounds originated from the initial electrodeposition step. The presence of Na 1s peak in Na30V@CC and other sodium-intercalated electrode materials corroborated the successful insertion of sodium ions into the vanadium oxide structure during the intercalation process. Furthermore, as shown in Table 1, the Na/V atomic ratios determined by both XPS and SEM-EDX analyses were generally consistent across all materials. The sodium content increased with prolonged intercalation time and approached a saturation level after 40 min.

Figure 4b illustrates the high-resolution XPS results for the O 1s and V 2p regions. In the Na30V@CC material, the V 2p peaks were deconvoluted into two prominent peaks corresponding to V 2p3/2 and V 2p1/2; each of these peaks was further split into four distinct components, occurring at binding energies of 515.9, 517.3, 518.8, and 524.4 eV. The peaks at 515.9 eV and 518.8 eV could be attributed to the presence of V^4+^, whereas the peaks at 517.3 eV and 524.4 eV were characteristic of V^5+^, suggesting a distinction between the two oxidation states [33,34]. The coexistence of two distinct valence states of vanadium indicated its dual valence, a crucial characteristic for its role as an active material in battery applications. Additionally, the oxygen peak (O 1s) was deconvoluted into two distinct peaks located at 529.9 eV and 531.7 eV. The peak at 529.9 eV was assigned to the vanadium oxide compound, while the peak at 531.7 eV corresponded to residual water molecules [35]. A similar analysis of the oxygen and vanadium high-resolution XPS spectra was also observed in other sodium intercalation electrode materials (Figure S13). Furthermore, the notable sodium peak (Na 1s, 1071 eV as illustrated in Figure 4c) observed subsequent to electrochemical intercalation served to validate the successful incorporation of sodium into the vanadium oxide matrix. Moreover, the intensity of the sodium peak (Na 1s) increased with the duration of sodium intercalation up to 40 min (Figure S14), suggesting that the sodium intercalation process reached saturation around this point. In summary, the XPS analysis corroborated that the initial electrochemical process resulted in the deposition of vanadium oxide on the surface of the carbon cloth. Also, the subsequent electrochemical reaction effectively facilitated the intercalation of sodium, which was consistent with the observations made through other characterization techniques.

**Figure 5:**
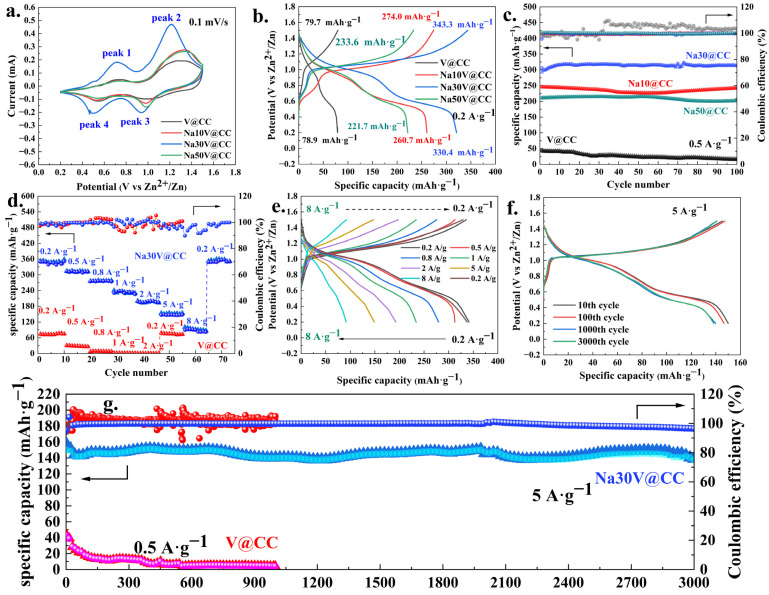
The electrochemical performance of zinc ion batteries (V@CC, Na10V@CC, Na30V@CC and Na50V@CC material): (**a**) CV scanning curves; (**b**) GCD curves (0.2 A·g^−1^); (**c**) preliminary cyclic performance at 0.5 A·g^−1^; (**d**) rate performance of 0.2 to 8 A·g^−1^ (V@CC and Na30V@CC materials); (**e**) GCD curves of Na30V@CC at different rate; (**f**) GCD curves of Na30V@CC after 10, 100, 1000, and 3000 cycles (5 A·g^−1^); (**g**) long cycle performance (5 A·g^−1^) of V@CC (1000) and Na30V@CC materials (3000). Notes: In (**c**,**g**), the triangular data points represent discharge capacity, while the circular data points represent charge capacity.

Figure 5 and Figures S15–S17 depict the electrochemical performance of the zinc-ion batteries. As shown in Figure 5a and Figure S15, the cyclic voltammetry (CV) curves of all materials displayed two distinct pairs of well-defined redox peaks, characteristic of the redox peaks of vanadium [36]. Notably, a comparative analysis revealed that the sodium-intercalated materials exhibited a substantially enhanced intensity of redox peaks in comparison to the V@CC. This phenomenon indicated that the introduction of sodium ions through intercalation significantly enhanced the electrochemical redox activity of the material. Among all sodium-intercalated electrode materials (Figure 5a and Figure S15), Na30V@CC exhibited the strongest oxidation-reduction peak intensity, indicating its superior electrochemical performance and suggesting its potential to deliver the best battery performance. Additionally, Figure 5b and Figure S16 displayed the charge–discharge (GCD) curves for all materials at a current density of 0.2 A·g^−1^. The GCD results revealed that the charge–discharge specific capacity of the pristine V@CC material was only 79.7 during charging and 78.9 mAh·g^−1^ during discharging, respectively. In contrast, sodium intercalation significantly enhanced the specific capacity of the batteries. All sodium-intercalated electrode materials exhibited higher charge–discharge specific capacities than the pristine V@CC material. Specifically, the Na30V@CC material exhibited the highest charge–discharge specific capacity, with values of 343.3 and 330.4 mAh·g^−1^, respectively. Excessive sodium insertion beyond 30 min would lead to the oversaturation of active sites, resulting in competitive ion diffusion with zinc ions. This competition would hinder efficient ion transport and ultimately contribute to a reduction in battery capacity [31]. Similar trends were observed in the preliminary cyclic performance tests (0.5 A·g^−1^), as shown in Figure 5c and Figure S17. Over the course of 100 cycles, the specific capacity of all materials, with the exception of V@CC, remained stable, with coulombic efficiency consistently approaching 100%. Meanwhile, the Na30V@CC electrode material exhibited the highest specific capacity, maintaining a value of approximately 300 mAh·g^−1^.

In addition, the evaluation of rate performance, a key parameter for determining battery efficiency, was conducted, with the corresponding results for V@CC and Na30V@CC presented in Figure 5d,e. As depicted in Figure 5d, the V@CC material exhibited relatively limited rate performance. When the charge–discharge current density exceeded 1 A·g^−1^, a significant reduction in specific capacity was observed. In comparison, the Na30V@CC material exhibited considerably superior rate performance, while its specific capacity decreased at a slower rate as the current density increased. Even at a charge–discharge current density of 8 A·g^−1^, it maintained a specific capacity of approximately 90 mAh·g^−1^. Throughout the entire testing process, although the coulomb efficiency fluctuates slightly during the high-rate charge and discharge process (1 to 8 A·g^−1^), overall, the fluctuation range of Na30V@CC material was significantly smaller than that of V@CC, and its coulomb efficiency basically remained above 90%. When the charge–discharge current density was reduced back to 0.2 A·g^−1^, the specific capacity of the Na30V@CC material rapidly recovered to approximately 340 mAh·g^−1^. The stability of coulombic efficiency under high-rate conditions will be one of the focuses of our subsequent research. Furthermore, long-term cycling stability was a critical criterion for the practical implementation of zinc-ion batteries, as it directly influenced their durability and operational reliability. Figure 5f,g present the long-cycle performance of V@CC and Na30V@CC materials. As observed, for the V@CC material, even at a lower charge–discharge current density of 0.5 A·g^−1^, its battery capacity exhibited a significant decline with increasing cycle number. After undergoing 1000 cycles, the specific capacity of V@CC material decreased to below 4 mAh·g^−1^, indicating a considerable loss in performance. On the other hand, following sodium intercalation, the Na30V@CC electrode material revealed exceptional stability. Even at a charge–discharge current density of 5 A·g^−1^, it exhibited only a slight decrease in capacity after 3000 cycles, with the coulombic efficiency maintaining stability at approximately 99%. Overall, the electrochemical performance results clearly demonstrated that sodium intercalation significantly enhanced the specific capacity, rate capability, and cycling stability of vanadium oxide, all of which were essential parameters for optimizing zinc-ion battery performance. Table S1 summarizes the recent advancements in the electrochemical performance of cathode materials for zinc-ion batteries. A comparative analysis of the data in Table S1 revealed that the Na30V@CC electrode material investigated in this study achieved a medium-to-high ranking in terms of both specific capacity and cycling stability.

**Figure 6:**
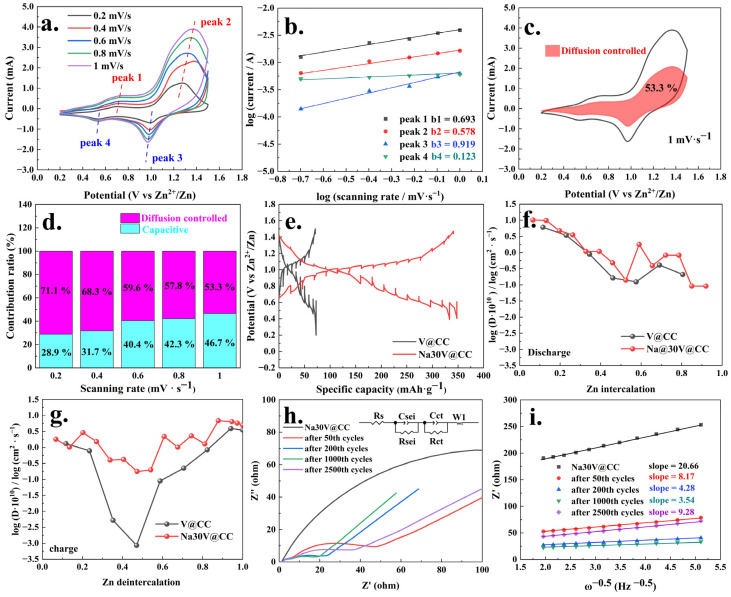
Dynamic behavior of Na30V@CC material: (**a**) CV scanning curves under different rate; (**b**) log(*i*) vs. log(*v*) plots of Na30V@CC material at main redox peak currents; (**c**,**d**) capacitive and diffusion contribution at 1 mV·s^−1^ and different scan rates; the GITT testing results of V@CC and Na30V@CC materials (**e**); the analysis of diffusivity coefficient (D) of Zn^2+^ in the discharge (**f**) and charge (**g**) processes of V@CC and Na30V@CC materials; the EIS curves and equivalent circuit model under different cycles for Na30V@CC material ((**h**): inserted picture is analog circuit) and analysis results of linear relationship between Z′ and ω^−0.5^ (**i**).

The investigation of dynamic behavior is essential for elucidating the intrinsic kinetic processes governing zinc-ion battery operation. To analyze these dynamic processes, the cyclic voltammetry (CV) measurements of the Na30V@CC electrode were conducted at various scan rates, as illustrated in Figure 6a. The CV curves distinctly exhibited two pairs of redox peaks, corresponding to the valence state transitions of peaks 1 (V^3+^ to V^4+^)/peaks 4 (V^4+^ to V^3+^) and peaks 2 (V^4+^ to V^5+^)/peaks 3 (V^5+^ to V^4+^). Notably, the intensity of the four redox peaks increased proportionally with the scan rate. Further analysis, employing Bruce Dunn’s dynamic process methodology [37,38], revealed a logarithmic linear relationship between the peak current and scan rate of the redox peaks, as described by Equations (1) and (2).

(1) $$I=av^{b}$$

(2) log(I)=blog(v)+log(a)

Here, *ν* corresponded to the scanning rate, *I* indicated the peak current, and a referred to the adjustable parameters in the equations. Formula (2) represented the logarithmic form of Formula (1).

Additionally, the results of the analysis using Bruce Dunn’s dynamic method were presented in Figure 6b. As illustrated in Figure 6b, the *b* values for the four peaks were 0.693, 0.578, 0.919, and 0.123, respectively. From a theoretical standpoint, a *b* value approaching 0.5 signified a diffusion-controlled process, while a *b* value near 1 indicated a surface-controlled process. Additionally, the experimental findings further indicated that the dynamic behavior of the zinc-ion battery incorporating Na30V@CC mainly involved both diffusion and surface control mechanisms. To determine the specific contributions of diffusion and surface control, both Formula (3) and its linearized form, Formula (4), were employed [39,40].

(3) $$i=k_{1}v+k_{2}v^{0.5}$$

(4) $$i/v^{0.5}=k_{1}v^{0.5}+k_{2}$$

Here, *ν* also corresponded to the scanning rate, and *I* indicated the peak current.

The data analysis results (Figure 6c,d) revealed that at a scan rate of 1 mV·s^−1^, the fraction of the contribution from diffusion-controlled processes reached its minimum value of 53.3%. At other scan rates, the diffusion-controlled contribution ratios were 71.1% (0.2 mV·s^−1^), 68.3% (0.4 mV·s^−1^), 59.6% (0.6 mV·s^−1^), and 57.8% (0.8 mV·s^−1^), as illustrated in Figures S18–S21. The remaining proportions, 28.9%, 31.7%, 40.4%, 42.3%, and 46.7%, corresponding to the surface-controlled processes, represented the pseudo-capacitive contributions. To gain a deeper understanding of the kinetics governing the battery reaction process, galvanostatic intermittent titration technique (GITT) testing [40] and analysis were performed on the V@CC and Na30V@CC materials, as presented in Figure 6e. The results demonstrated that throughout the GITT testing, both batteries consistently retained their respective capacities, with the Na30V@CC material exhibiting a significantly higher capacity than the V@CC, underscoring its superior electrochemical performance. Moreover, the analysis of the zinc diffusion coefficients, as presented in Figure 6f,g, revealed significant differences between the Na30V@CC and V@CC materials during the charging process. For the Na30V@CC material, the zinc deintercalation diffusion coefficient consistently ranged between approximately 10^−10^ to 10^−11^ cm^2^·s^−1^. In comparison, the diffusion coefficient for the V@CC material exhibited a wider range, spanning from approximately 10^−10^ to 10^−14^ cm^2^·s^−1^. These findings clearly demonstrated that the zinc deintercalation diffusion coefficient in the Na30V@CC electrode material was substantially higher than that of the V@CC material. Conversely, during the discharge process, the diffusion coefficients of zinc intercalation for both materials were within the range of approximately 10^−10^ to 10^−11^ cm^2^·s^−1^, with the Na30V@CC electrode material demonstrating a slightly higher diffusion curve compared to the V@CC material. In summary, the GITT analysis results underscored that sodium intercalation significantly enhanced the zinc diffusion kinetics throughout the battery reaction during both the charging and discharging processes.

The evolution of the electrochemical impedance spectroscopy (EIS) spectra during the cycling process for the Na30V@CC and V@CC materials was subsequently analyzed, with the results depicted in Figure 6h and Figure S22. As illustrated, the impedance spectra displayed features characteristic of conventional battery reaction behavior. A quantitative analysis of these spectra was conducted using a theoretical equivalent circuit model (inset), which served as a representative framework for interpreting battery impedance responses. As presented in Table 2, the internal resistance (Rs) of the Na30V@CC battery system exhibited minimal fluctuations throughout the entire cycle, ranging from 1.669 to 1.887 Ω. This consistency indicated the structural integrity of the battery. In contrast, the charge transfer resistance (Rct) exhibited more pronounced variations, underscoring its sensitivity to the cycling process. Throughout the cycling process, from the initial state to 1000 cycles, the charge transfer resistance exhibited a progressive reduction, with values of 195.9, 45.16, 22.02, and 16.55 Ω, respectively. Furthermore, at every stage of the reaction, the internal resistance (Rs) and charge transfer resistance (Rct) of the V@CC material were consistently higher compared to those of the Na30V@CC material. These findings highlighted the advantageous effect of sodium intercalation in improving the kinetics of the battery reaction.

The analysis of both the EIS curve trends and the corresponding data revealed that, as the charge–discharge cycles progressed, the charge transfer resistance progressively decreased, indicating increasingly efficient and smoother battery reactions. For the Na30V@CC electrode material, a gradual increase in charge transfer resistance Rct to 36.41 Ω was observed after 2500 cycles, suggesting a decline in battery performance during the later stages of cycling. This deterioration was likely attributed to subtle structural damage to the button battery pack. In comparison, the V@CC material exhibited a substantial increase in impedance, reaching 982.4 Ω after only 1000 cycles (Table 2), underscoring its considerably lower electrochemical stability relative to the Na30V@CC electrode. The trends observed in the charge transfer resistance in Na30V@CC were also reflected in the variations in the semicircular arc diameter of the electrode, which corresponded to the value of electrolyte resistance (Rsei). In addition, the zinc ion diffusion coefficient was determined by analyzing the linear relationship between the real part of the impedance (Z′) and the angular frequency (ω^−0.5^) in the relevant section of the impedance spectrum [41], as depicted in Figure 6i. The slopes of the linear fits for the different stages of cycling were determined to be 20.66 (pristine), 8.17 (50th), 4.28 (200th), 3.54 (1000th), and 9.28 (2500th). Since the square of the slope was inversely proportional to the zinc ion diffusion coefficient [42], these values indicated that the zinc ion diffusion rate for the Na30V@CC electrode material was significantly higher than that in the V@CC material (Figure S23). Furthermore, the specific values of the apparent zinc ion diffusion coefficient were calculated using equations S1 and S2, with the corresponding results summarized in Table S2. From this comparison, it was evident that the diffusion coefficient values derived from both methods, EIS analysis and GITT testing (Figure 6f,g), were within the same order of magnitude (approximately 10^−10^ cm^2^·s^−1^). Considering the inherent differences and potential sources of error associated with each testing technique, these results were deemed consistent and acceptable for characterizing the zinc-ion diffusion behavior during the battery reaction process. Moreover, the zinc ion diffusion rate exhibited an initial increase followed by a subsequent decrease during the battery cycling process, a trend that mirrored the behavior of the charge transfer resistance.

**Figure 7:**
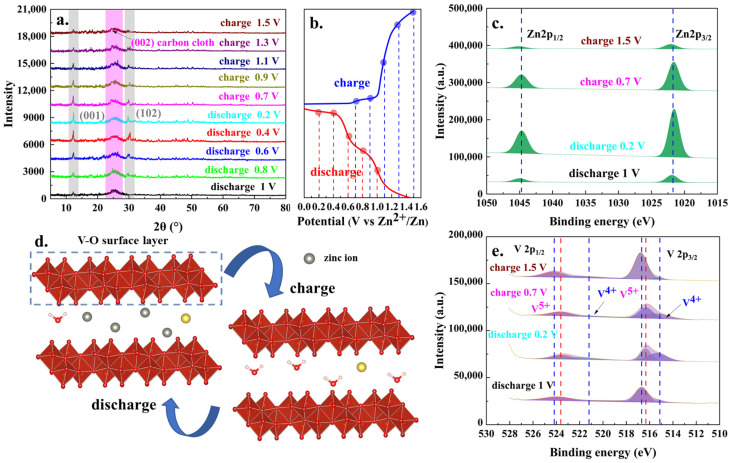
The XRD patterns during a discharge–charge cycle (**a**); the corresponding GCD curve (**b**); the high-resolution Zn2p (**c**) and V2p (**e**) analysis results of charge-1.5V, charge-0.7V, charge-0.2V, and charge-1V statuses; the zinc injection and removal processes during charging and discharging process (**d**).

Building upon previous studies, the mineral phase evolution and changes in elemental valence states during the charge and discharge processes were systematically investigated using XRD and XPS. As depicted in Figure 7a, the most pronounced XRD peak shifts were observed at the 001 crystal plane, located at 12.2° (2θ). By correlating these changes with the corresponding galvanostatic charge–discharge (GCD) curve, it was observed that during the discharge process, the intensity of the 001 crystal plane peak gradually increased, while during the charge process, the peak intensity exhibited a reverse trend. Similar phenomena had been frequently observed in the charge and discharge processes of various vanadium-based zinc-ion batteries. The increase in intensity of the 001 crystal plane could be attributed to the continuous insertion of zinc ions into the V-O surface layer of vanadium oxide during the discharge process. Conversely, during the charging process, the progressive extraction of zinc ions from the V–O layers led to a corresponding decrease in the intensity of the 001 crystal plane peak. This process was further corroborated by the XPS full spectrum scanning analysis (Figure S24) and high-resolution Zn 2p spectrum (Figure 7c), which revealed that the intensities of the Zn 2p_3/2_ and Zn 2p _1/2_ peaks at discharge (−0.2 V) and charge (−0.7 V) states were substantially stronger than those observed at discharge (−1.0 V) and charge (−1.5 V) states. Furthermore, Figure 7e presents the high-resolution V 2p XPS analysis for the discharge (−0.2 V), discharge (−1.0 V), charge (−0.7 V), and charge (−1.5 V) states. Overall, the peak intensities of the vanadium species across these four states remained relatively stable, indicating that the structural integrity of the vanadium oxide framework was preserved throughout the entire charge and discharge cycle. The variations in the relative intensities of the V^4+^ and V^5+^ peaks corresponded to the reduction and oxidation processes of the vanadium species during the discharge and charge cycles, respectively.

**Figure 8:**
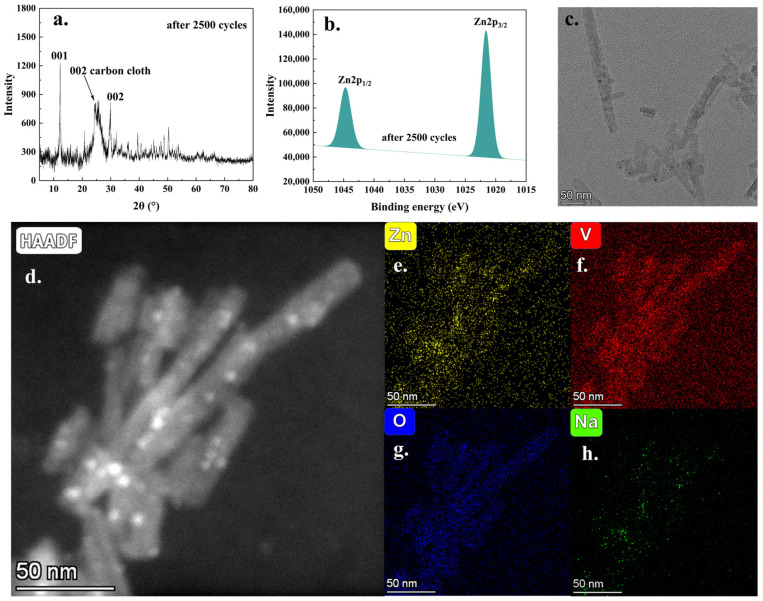
The XRD pattern (**a**); the high-resolution Zn2p analysis result (**b**); TEM (**c**,**d**); and EDX element mapping analysis results (**e**–**h**) after 2500 charge–discharge cycles.

Furthermore, Figure 8 presents the characterization results of the battery reaction products after 2500 charge–discharge cycles. As depicted in Figure 8a, the principal mineral phases of the battery reaction products exhibited stability following the 2500th discharge cycle. Additionally, the XPS analysis (Figure S25) verified the presence of vanadium, oxygen, zinc, and trace amounts of sodium in the reaction products. The high-resolution Zn 2p spectrum (Figure 8b) exhibited an enhanced intensity of the Zn 2p peak, which aligned with the anticipated reaction processes occurring during the battery cycling. In addition, the presence of a strong V 2p peak (Figure S26) suggested that, after 2500 charge–discharge cycles, the vanadium oxide framework remained structurally stable. TEM analysis (Figure 8c,d) illustrated that the nanorod morphology persisted, although the original nanowire structure appeared to be disrupted and shortened due to the frequent charge–discharge cycles. TEM-EDX elemental analysis (Figure 8e–h) further confirmed the presence of significant amounts of zinc, vanadium, and oxygen elements in the analysis area, with only trace levels of sodium detected. These findings were consistent with the XPS results and supported the proposed reaction mechanism. In summary, after prolonged cycling, the battery’s active material retained its nanoscale structural morphology and exhibited stable mineral phase and elemental composition, which were critical for maintaining long-term electrochemical performance. Based on the comprehensive data and analyses presented above, it could be concluded that the cathode material (NaXV@CC), suitable for application in aqueous zinc-ion batteries, was successfully synthesized via a two-step electrochemical approach involving deposition and sodium intercalation. The electrochemical intercalation of sodium ions could significantly alter the interplanar spacing of the (001) crystal plane and the material’s micromorphology, thereby effectively enhancing the efficiency of zinc-ion insertion, extraction, and diffusion during the charge–discharge process. These advantageous effects enabled the assembled battery to achieve a high specific capacity exceeding 330.4 mAh·g^−1^ at a current density of 0.2 A·g^−1^, with stable cycling performance maintained over 3000 cycles at 5 A·g^−1^. Therefore, the two-step electrochemical method can be regarded as an effective and practical strategy for synthesizing high-performance cathode materials for aqueous zinc-ion batteries.

## 3. Experiments and Methods

The chemical reagents used in this experiment were purchased from Sigma Reagents Company and were of analytical grade, used without further purification. The carbon cloth (CC) employed in this study was supplied by Suzhou Sinero Technology Co., Ltd., (Kunshan, China) with the following initial specifications: thickness of 0.35 mm, nominal basis weight of 140 g/m^2^, Gurley air permeability of 1.56 s, and plane resistance of 1.50 mΩ/cm^2^. Prior to use, the carbon cloth was subjected to heat treatment at 450 °C for two hours, followed by gradual cooling to room temperature. This treatment was carried out to enhance the hydrophilicity of the carbon cloth, thereby improving its suitability as a conductive substrate for electrochemical applications (Figure S27).

The V@CC material was synthesized through an electrodeposition technique. The electrolyte employed in the process was a 0.1 M vanadyl sulfate (V[IV]OSO_4_) solution. A conventional three-electrode system was employed, in which the treated hydrophilic carbon cloth (1.5 × 1.5 cm) functioned as the working electrode, a platinum wire served as the counter electrode, and an Ag/AgCl electrode was used as the reference electrode [43]. The electrodeposition was performed in a temperature-controlled water bath maintained at 65 °C, with an applied potential of 1.2 V for the duration of 20 min. The resulting V@CC material was air-dried under ambient conditions for subsequent application.

The loading amount of active vanadium oxide ranged from approximately 2 mg to 2.5 mg, as determined by the mass difference in the carbon cloth before and after the deposition process. The mass of active material obtained here was used to calculate the charge/discharge specific capacity of the battery. Accordingly, the mass ratio of active material to the carbon cloth substrate was approximately in the range of 1:8.5 to 1:6.8. To facilitate material characterization, the active substance on the V@CC material was separated via ultrasonic treatment and subsequently identified as “vanadium oxide”.

Sodium ion intercalation was achieved through an electrochemical process utilizing a two-electrode system (electrolyte was 0.1 mol/L NaCl solution). The prepared V@CC material was employed as the working electrode, with a zinc sheet functioning as both the counter and reference electrode. The electrochemical intercalation was performed at a constant voltage of 1.2 V for varying durations of 10, 20, 30, 40, 50, and 60 min. Following the intercalation process, the materials were air-dried under ambient conditions and designated as Na10V@CC, Na20V@CC, Na30V@CC, Na40V@CC, Na50V@CC, and Na60V@CC, corresponding to the respective intercalation times. The amount of sodium-intercalated vanadium oxide loaded onto the material ranged from approximately 2.5 mg to 3 mg, as determined by the mass difference between the modified samples and the original carbon cloth. The mass of active material obtained here was used to calculate the charge/discharge specific capacity of the battery. Accordingly, the mass ratio of active material to the carbon cloth substrate was approximately 1:6.8 to 1:5.67. For characterization purposes, the active substance on the Na30V@CC was separated using ultrasonic treatment and subsequently identified as “Na30-vanadium oxide”.

A variety of advanced analytical instruments were applied to thoroughly characterize the synthesized materials. The mineral phase composition of materials was investigated through X-ray diffraction (XRD), which was recorded using a Beijing Puxi XD-6 polycrystalline X-ray diffractometer (Laiwu, China) with Cu-Kα radiation (λ = 1.54056 Å). In order to ensure the accuracy of the test, all powder materials were ground through a 400-mesh sieve, and flake materials were used directly. The scanning step length was 0.02° and the scanning rate was 1°/min. Scanning electron microscopy (SEM) and transmission electron microscopy (TEM) were employed to investigate the morphological features and microelement composition of the materials. The detailed information of instruments included a HITACHI Regulus 8100 SEM integrated with a Super-X EDS detector (Beijing, China), and a FEI Talos F200s TEM, which included high-angle annular dark-field scanning transmission electron microscopy (HAADF-STEM) and a FEI Super-X EDS detector (Thermo Fisher Scientific, Shanghai, China). The accelerating voltage for TEM testing was 200 kV. The elemental composition and chemical states were analyzed through X-ray photoelectron spectroscopy (XPS) measurements carried out with a PHI 5000 VersaProbe instrument (Physical Electronics, Chanhassen, MN, USA).

**Figure 9:**
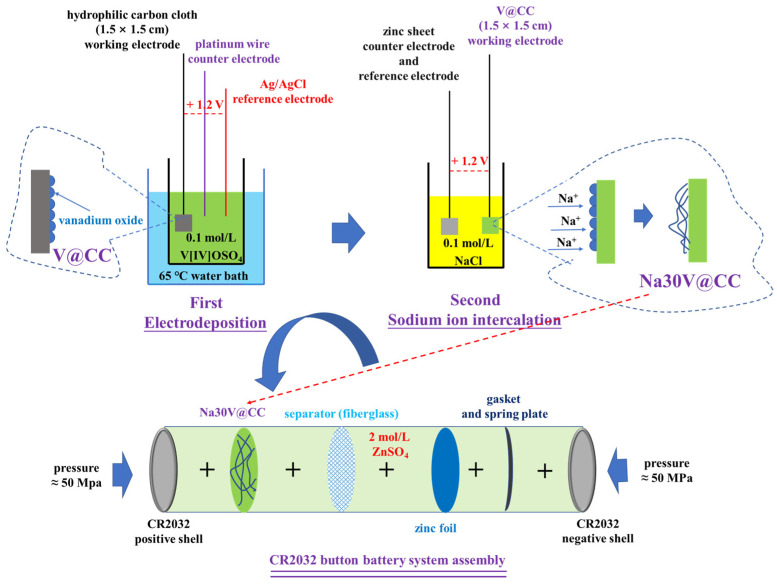
The schematic diagram of the material synthesis and battery assembly process.

The electrochemical properties of the synthesized materials were evaluated by assembling zinc-ion button-type batteries (CR2023), with a 2 M zinc sulfate solution as the electrolyte and a GF/A fiberglass membrane serving as the battery separator. The cathode was made from the synthesized materials, while a circular zinc sheet (13 mm in diameter and 0.1 mm in thickness) acted as the anode. The battery was assembled in a particular order, starting with the positive shell, followed by the cathode material, separator, anode, gasket, spring plate, and ending with the negative shell. The quality of each part was approximately: 0.887 g (positive shell), 0.0195 to 0.02 g (cathode material), 0.0368 g (separator), 0.0923 g (Zn foil anode), 1.4462 g (gasket), 0.363 g (spring plate), and 0.855 g (negative shell). A compression pressure of 50 MPa was applied during the assembly process to ensure proper contact between the components and maintain the structural integrity of the components. The schematic illustration of the material synthesis and battery assembly process is described in Figure 9.

The evaluation of zinc-ion battery performance encompassed cyclic voltammetry (CV), electrochemical impedance spectroscopy (EIS), galvanostatic charge–discharge (GCD) testing, and rate performance analysis. In addition, both preliminary and extended cycle testing are conducted. CV and EIS measurements were performed using an electrochemical workstation (Shanghai Chenghua 660E (Shanghai Chenghua Instruments Inc., Shanghai, China)).

The CV tests were performed within a voltage range of 0.2 to 1.5 V, at scan rates of 0.1, 0.2, 0.4, 0.6, 0.8, and 1 mV·s^−1^. EIS measurements were carried out with an amplitude of 0.005 V, covering a frequency range of 0.01 to 100,000 Hz. All other battery performance tests were executed using the Land battery testing system. In addition, current densities of 0.2, 0.5, 0.8, 1, 2, 5, and 8 A·g^−1^ were applied for GCD and rate performance tests. The preliminary cycle testing involved 100 GCD cycles at a current density of 0.5 A·g^−1^, while the long-cycle stability was assessed over 3000 GCD cycles at 5 A·g^−1^. The specific capacity is calculated through dividing the battery charge/discharge capacity (measured by the instrument) by the amount of active material loaded on the surface of the carbon cloth.

## 4. Conclusions

In summary, the designed electrode materials of NaXV@CC were employed as a cathode for AZIBs in this work. Sodium ions were successfully intercalated into the nanowire lattice, resulting in notable changes in the overall nanostructure, as evidenced by a transformed surface morphology. In addition, the sodium content increased with prolonged intercalation time and approached a saturation level after 40 min. Among the electrode materials with varying intercalation durations, Na30V@CC demonstrated the most favorable electrochemical activity, characterized by high specific capacity, outstanding rate capability, and exceptional cycling stability. The unique nanowire-like morphology of Na30V@CC, featuring nanoscale wrinkles, offered a high density of active sites and facilitated efficient ion transport pathways, thereby enhancing its electrochemical performance. At a current density of 0.2 A·g^−1^, the electrode delivered charge and discharge capacities of 343.3 and 330.4 mAh·g^−1^, respectively. Impressively, the electrode sustained a capacity of nearly 90 mAh·g^−1^ at an elevated current density of 8 A·g^−1^, highlighting its excellent rate capability. In addition, the Na30V@CC electrode material exhibited exceptional stability, showing only a minor decline in capacity after 3000 charge–discharge cycles, while maintaining a coulombic efficiency of approximately 99% throughout the cycling process. Sodium intercalation significantly enhanced the specific capacity, rate capability, and cycling stability of vanadium oxide, all of which were essential parameters for optimizing zinc-ion battery performance. Both diffusion and surface-controlled processes contributed to the reaction dynamics, with the diffusion-dominated contribution increasing from 53.3% at 1 mV·s^−1^ to 71.1% at 0.2 mV·s^−1^. Furthermore, Na30V@CC, when applied as the cathode material for AZIBs, demonstrated reduced electrochemical impedance and accelerated zinc-ion diffusion rate, contributing to its superior electrochemical characteristics.

## Figures and Tables

**Table 1:** The ratio of sodium to vanadium (Na/V) on the surface of the composite material was determined by SEM-EDX and XPS analysis.

Sample	SEM-EDX	XPS
V@CC	0	0
Na10V@CC	-	≈0.211:1
Na20V@CC	≈0.36:1	≈0.331:1
Na30V@CC	≈0.465:1	≈0.442:1
Na40V@CC	≈0.57:1	≈0.562:1
Na50V@CC	-	≈0.564:1
Na60V@CC	-	≈0.566:1

Note: “-” means data not available.

**Table 2:** The impedance spectrum fitting and analysis results of Na30V@CC material.

Material	Rs (ohm)	Rsei (ohm)	Rct (ohm)
Pristine Na30V@CC	1.887	-	195.9
After 50th cycles (5 A·g^−1^)	1.678	5.694	45.16
After 200th cycles	1.669	5.809	22.02
After 1000th cycles	1.827	5.427	16.55
After 2500th cycles	1.841	6.072	36.41
Pristine V@CC	2.01	-	290.4
After 50th cycles (0.5 A·g^−1^)	2.09	-	123.2
After 200th cycles	2.15	-	105.2
After 500th cycles	2.19	-	87.7
After 1000th cycles	2.28	-	982.4

## Data Availability

Data are contained within the article and Supplementary Materials.

## Supplementary Materials

The following supporting information can be downloaded at: https://www.mdpi.com/article/10.3390/molecules30092074/s1, Figure S1: The comparison of actual XRD patterns of vanadium oxide and theoretically calculated result of crystal model after previous DFT calculations (insert picture: the bigger red ball represents vanadium atom, and the smaller red ball represents oxygen atom); Figure S2: The theoretically calculated result of V_2_O_5_·H_2_O crystal model after previous DFT calculations; Figure S3: The XRD patterns of Na10V@CC, Na20V@CC, Na30V@CC, Na40V@CC, Na50V@CC and Na60V@CC electrode materials; Figure S4: The SEM analysis results of original carbon cloth and V@CC; Figure S5: The SEM-EDX element mapping analysis results of area of Figure 2b; Figure S6: The SEM-EDX element mapping analysis results of area of Figure 3b; Figure S7: The SEM analysis results of Na20V@CC (a) and Na40V@CC (b) materials; Figure S8: The SEM-EDX element composition analysis results of area of Figure S7a; Figure S9: The SEM-EDX element composition analysis results of area of Figure S7b; Figure S10: The SEM-EDX element mapping analysis results of area of Figure S7a; Figure S11: The SEM-EDX element mapping analysis results of area of Figure S7b; Figure S12: The XPS full spectrum scanning of Na10V@CC, Na20V@CC, Na30V@CC, Na40V@CC, Na50V@CC and Na60V@CC materials; Figure S13: The High-resolution XPS results of O 1s and V2p of Na10V@CC, Na20V@CC, Na30V@CC, Na40V@CC, Na50V@CC and Na60V@CC electrode materials; Figure S14: The High-resolution XPS results of Na1s in Na10V@CC, Na20V@CC, Na30V@CC, Na40V@CC, Na50V@CC and Na60V@CC electrode materials; Figure S15: The electrochemical CV scanning curves of Na20V@CC, Na40V@CC and Na60V@CC materials; Figure S16: The GCD curves (0.2 A·g^−1^) of Na20V@CC, Na40V@CC and Na60V@CC materials; Figure S17: The preliminary cyclic performance at 0.5 A·g^−1^ of Na20V@CC, Na40V@CC and Na60V@CC materials; Figure S18: The capacitive and diffusion contribution at 0.2 mV·s^−1^; Figure S19: The capacitive and diffusion contribution at 0.4 mV·s^−1^; Figure S20: The capacitive and diffusion contribution at 0.6 mV·s^−1^; Figure S21: The capacitive and diffusion contribution at 0.8 mV·s^−1^; Figure S22: The EIS curves and equivalent circuit model under different cycles for V@CC material (h: inserted picture is analog circuit); Figure S23: The analysis results of linear relationship between Z′ and ω^−0.5^ of V@CC material; Figure S24: The XPS full spectrum scanning result of charge-1.5V, charge-0.7V, charge-0.2V and charge-1V statuses; Figure S25: The XPS full spectrum scanning result after 2500 charge-discharge cycles; Figure S26: The high-resolution V2p analysis result after 2500 charge-discharge cycles; Figure S27: The digital photos of hydrophilicity testing of original carbon cloth and carbon cloth after heat treatment; Table S1: The summary of recent electrochemical performance of cathode materials in ZIBs; Table S2: The Zinc ion diffusion coefficient obtained from low-frequency range of EIS curves. References [44,45,46,47,48,49,50,51,52,53,54,55,56,57,58,59] are cited in the Supplementary Materials.
