# Supplementary material for: Sodium-Intercalated Vanadium Oxide Coated on Carbon Cloth for Electrode Materials in High-Performance Aqueous Zinc-Ion Batteries
Source: Molecules. 2025 May 7;30(9):2074. doi: 10.3390/molecules30092074 (PMC12073733; doi:10.3390/molecules30092074)
Supplement: Supplementary file 1 [file molecules-30-02074-s001.zip › molecules-3565631-supplementary.pdf]

Supplementary Materials

Sodium-Intercalated Vanadium Oxide Coated on Carbon Cloth for Electrode Materials in High-Performance Aqueous Zinc-Ion Batteries

Chen Chen[1] *, Baoxuan Hou[1], Ting Cheng[1,2], Fei Wu[1], Yulin Hu[3], Youzhi Dai[4], Xiao Zhang[5], Yuan Tian[1], Xin Zhao[1], Lei Wang[1]

[1] School of Environmental and Chemical Engineering, Jiangsu University of Science and Technology, Zhenjiang, 212100, China;

[2] School of Environmental Ecology, The City Vocational College of Jiangsu, Nanjing 210017, China;

[3] College of Chemistry and Chemical Engineering, Anshun University, Anshun 561000, China;

[4] College of Environment and Resource, Xiangtan University, Xiangtan 41105, China;

[5] Nanjing University and Yancheng Academy of Environmental Technology and Engineering, Yancheng 224000, China

* Chen Chen (ORCID: 0000-0003-3714-7076); Email: chenc@just.edu.cn

Tel.: +86-0511-85639001; Fax: +86-0511-85639001

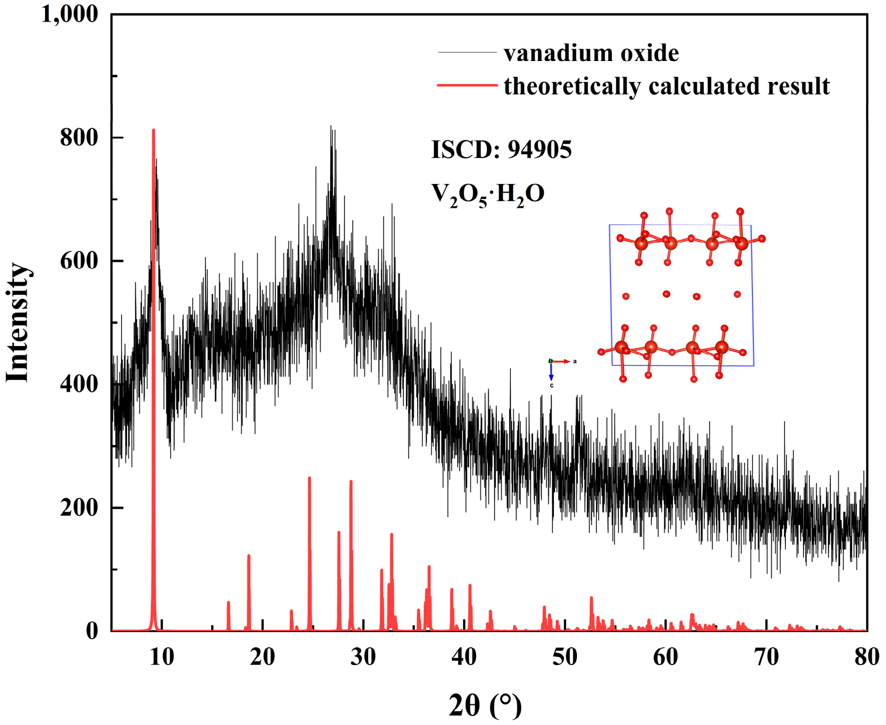

Fig.S1 The comparison of actual XRD patterns of vanadium oxide and theoretically calculated result of crystal model after previous DFT calculations (insert picture: the bigger red ball represents vanadium atom, and the smaller red ball represents oxygen atom)

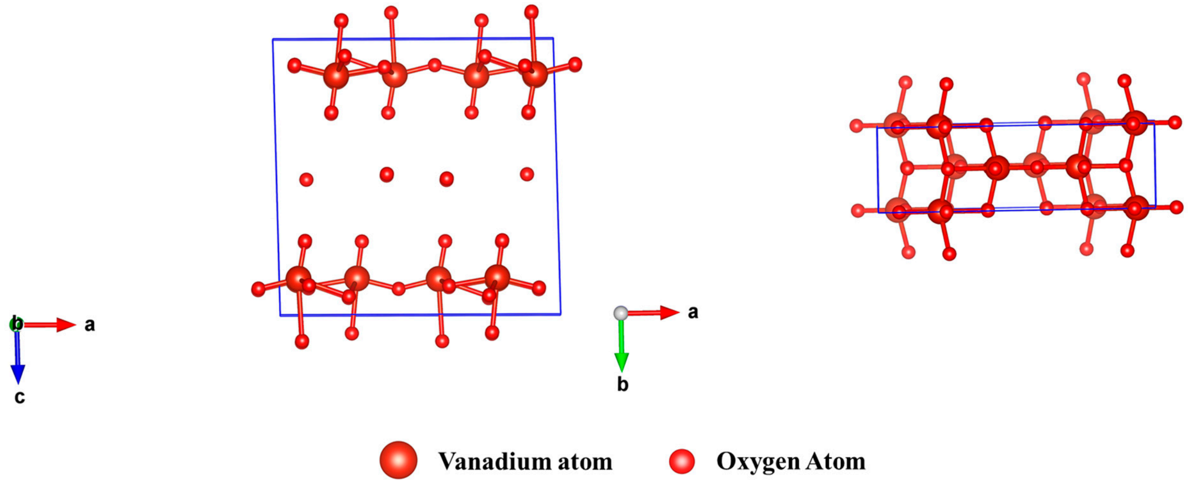

Fig.S2 The theoretically calculated result of $V_2O_5 \cdot H_2O$ crystal model after previous DFT calculations

In this study, the structural optimization and electronic property calculations of $V_2O_5 \cdot H_2O$ were conducted using spin-polarized Density Functional Theory (DFT) within the Vienna Ab initio Simulation Package (VASP). The exchange-correlation potential was determined using the Perdew-Burke-Ernzerhof functional (PBE) within the generalized gradient approximation (GGA) method. The cut-off energy was set at 550 eV, and the K-points grids of dimensions were 5×5×5 during the computation. The convergence criterion for energy was set at 10^{-5} eV. Fig.S1 illustrate the theoretical unit cell of $V_2O_5 \cdot H_2O$. The theoretical cell structure comprised 12 Vanadium atom, and 36 oxygen atoms. The space group was C2/m and the cell constants were 11.722 Å (a), 3.57 Å (b), 11.52 Å (c), 90.0 ° (α), 88.65 ° (β) and 90 ° (γ).

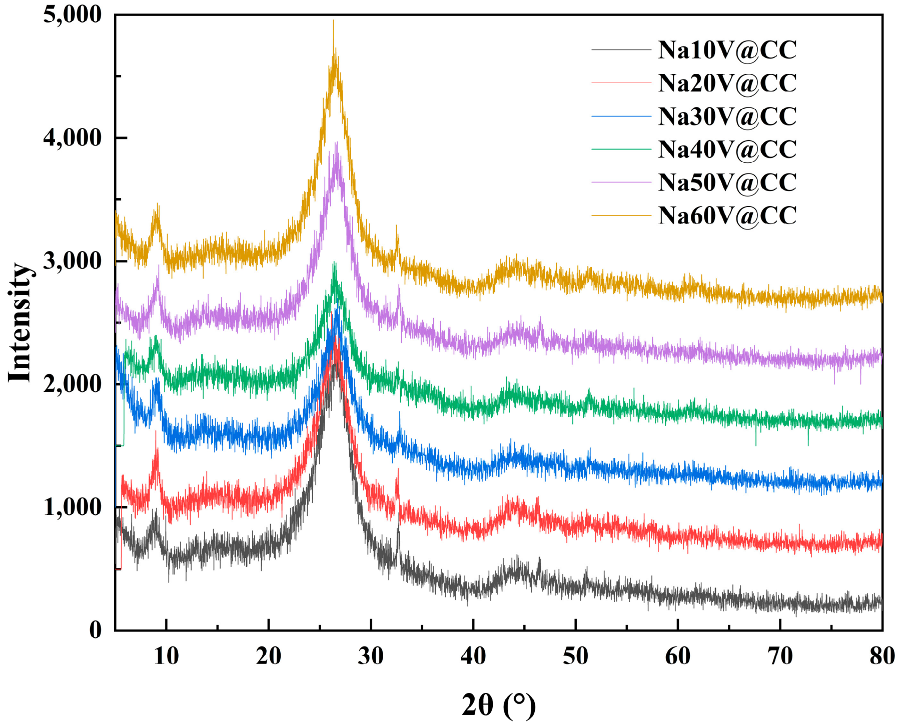

Fig.S3 The XRD patterns of Na10V@CC, Na20V@CC, Na30V@CC, Na40V@CC, Na50V@CC and Na60V@CC electrode materials

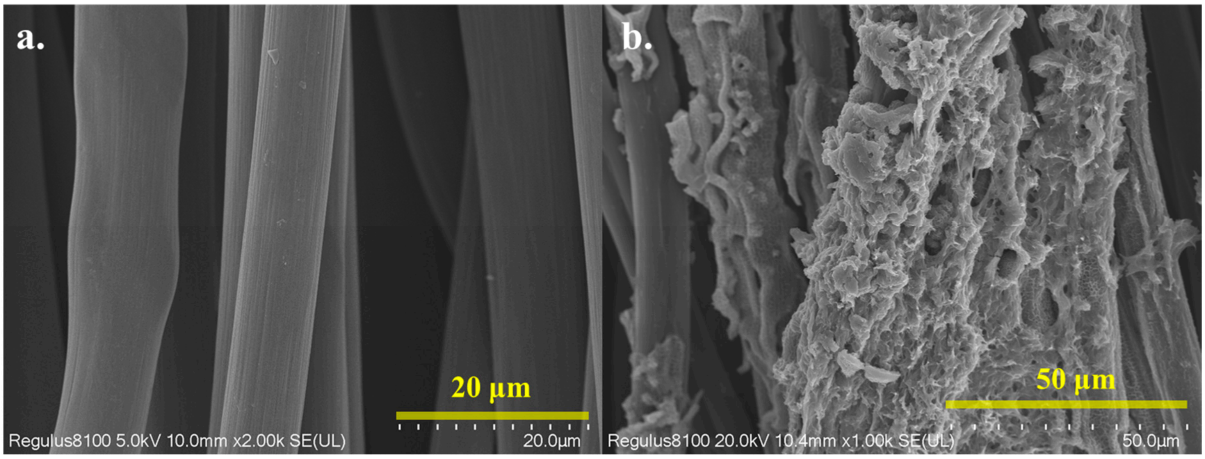

Fig.S4 The SEM analysis results of original carbon cloth and V@CC

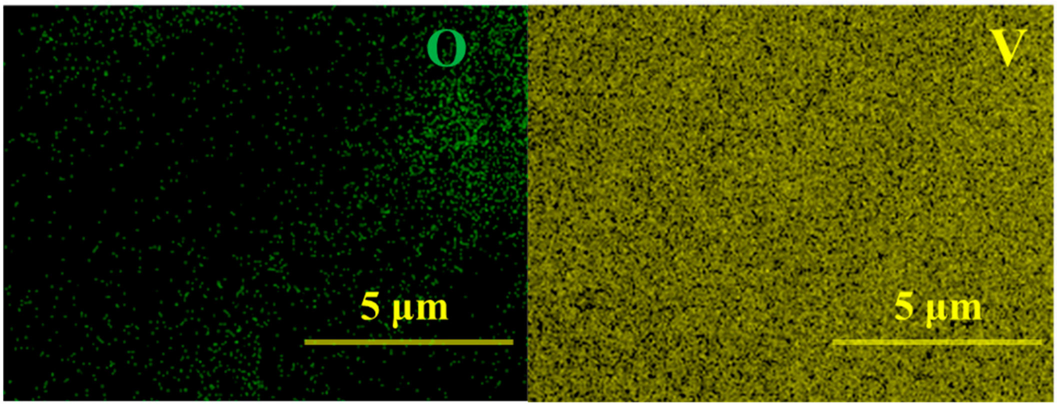

Fig.S5 The SEM-EDX element mapping analysis results of area of Fig.2b

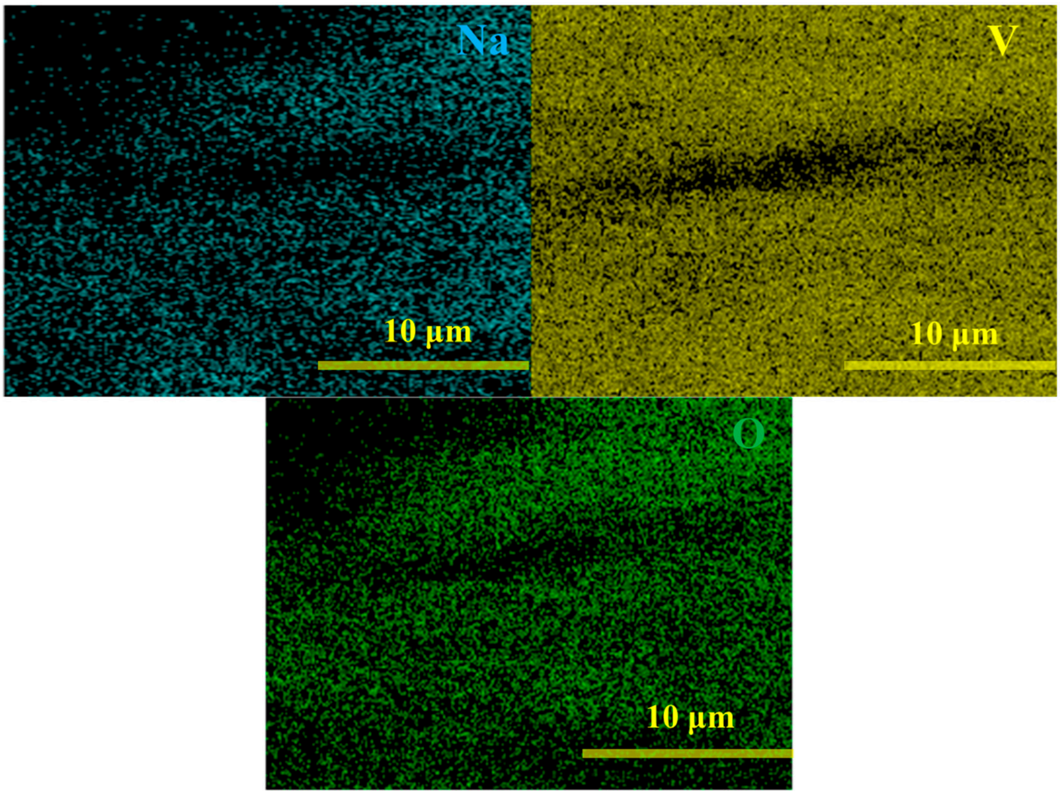

Fig.S6 The SEM-EDX element mapping analysis results of area of Fig.3b

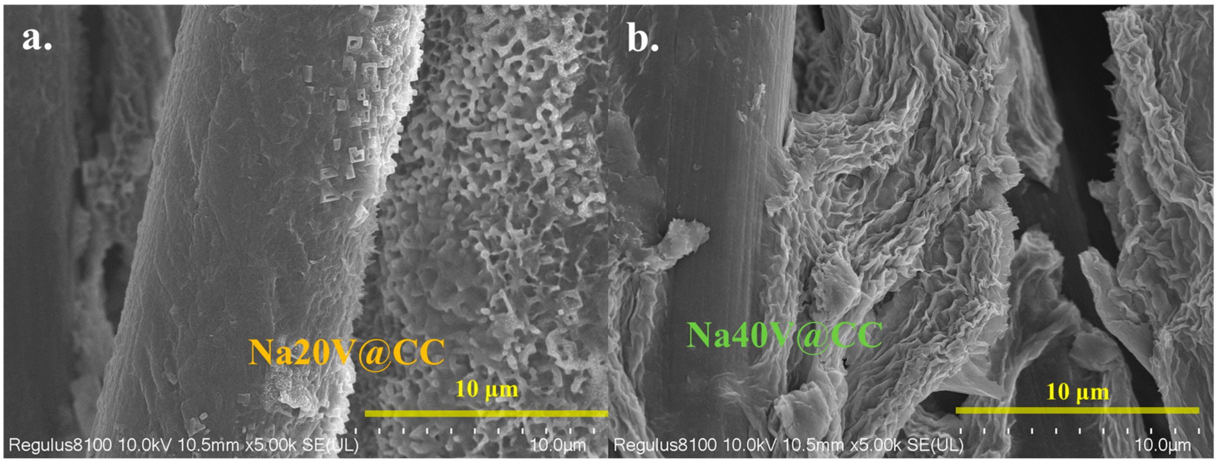

Fig.S7 The SEM analysis results of Na20V@CC (a) and Na40V@CC (b) materials

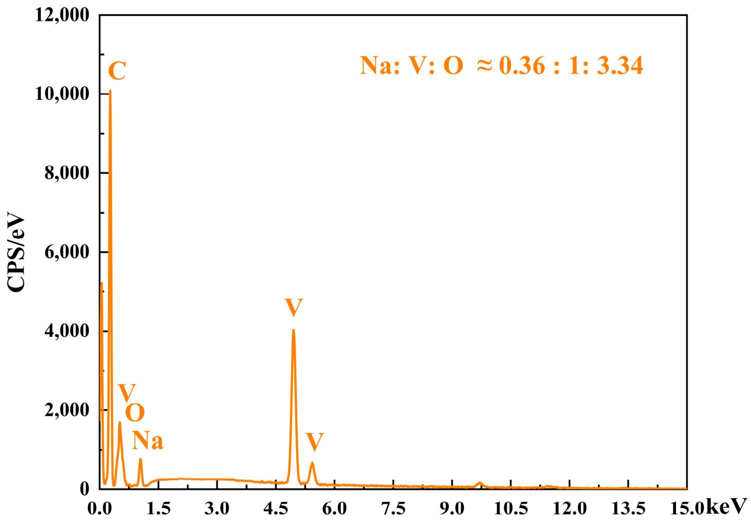

Fig.S8 The SEM-EDX element composition analysis results of area of Fig.S7a

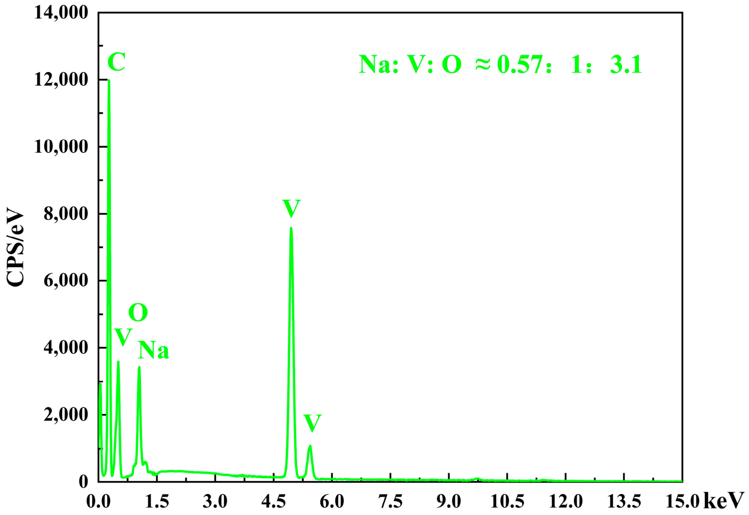

Fig.S9 The SEM-EDX element composition analysis results of area of Fig.S7b

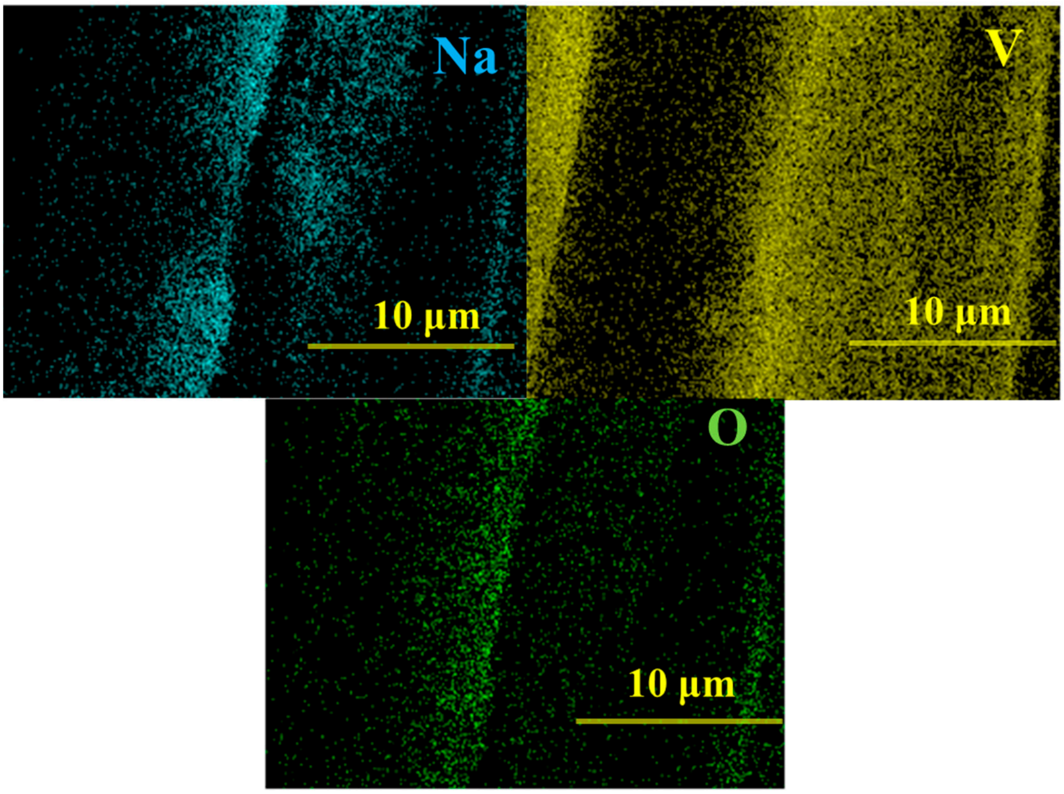

Fig.S10 The SEM-EDX element mapping analysis results of area of Fig.S7a

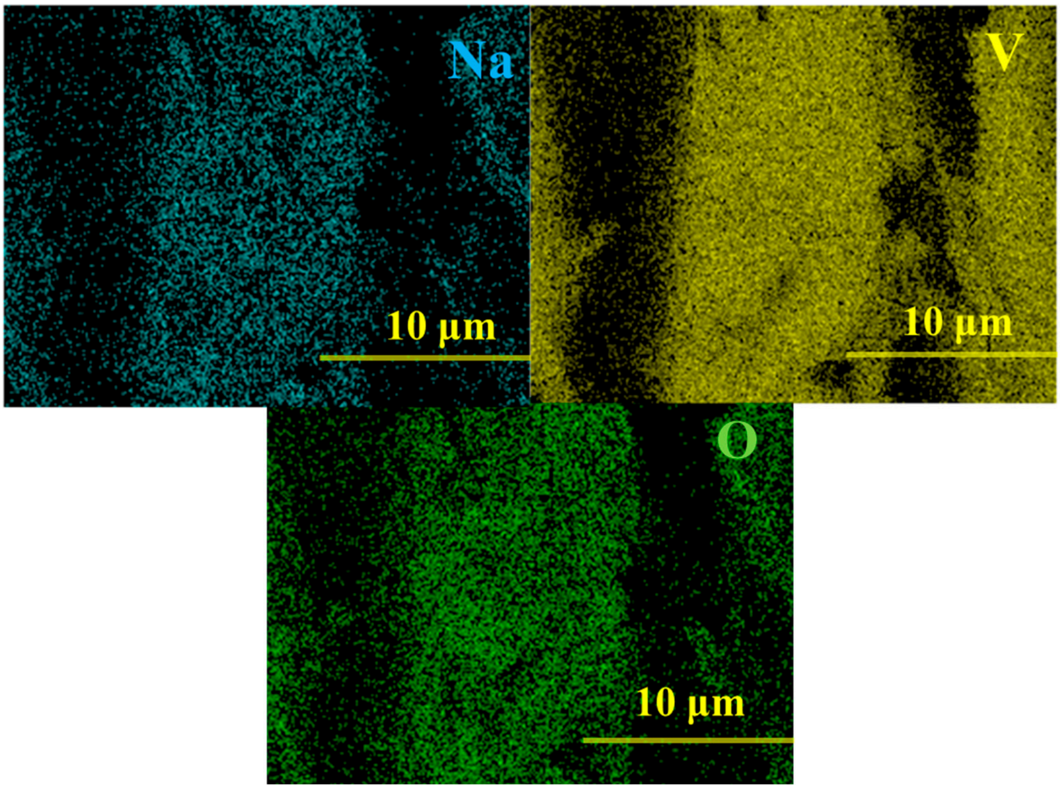

Fig.S11 The SEM-EDX element mapping analysis results of area of Fig.S7b

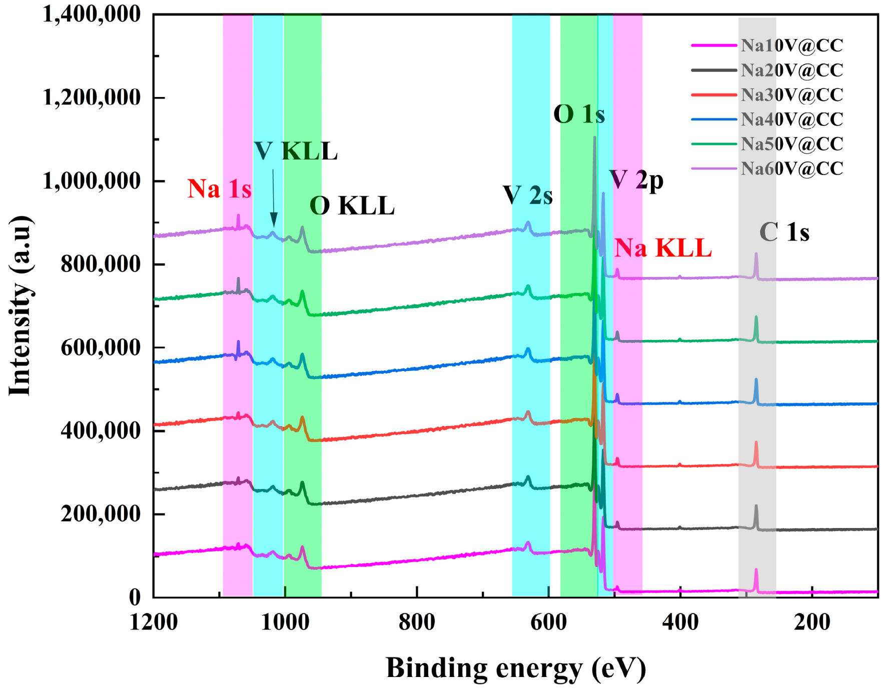

Fig.S12 The XPS full spectrum scanning of Na10V@CC, Na20V@CC, Na30V@CC, Na40V@CC, Na50V@CC and Na60V@CC materials

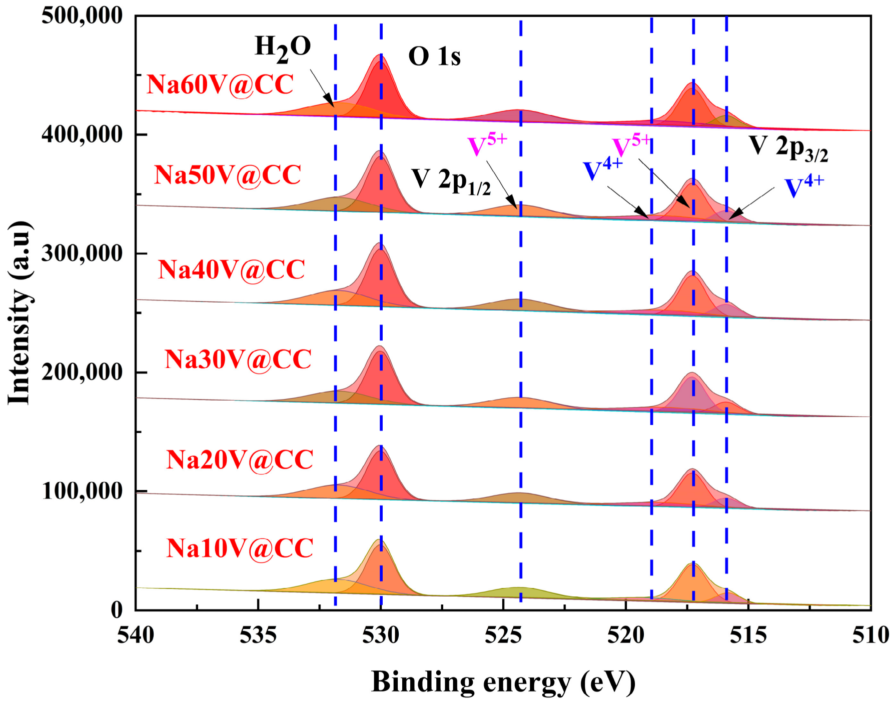

Fig.S13 The High-resolution XPS results of O 1s and V2p of Na10V@CC, Na20V@CC, Na30V@CC, Na40V@CC, Na50V@CC and Na60V@CC electrode materials

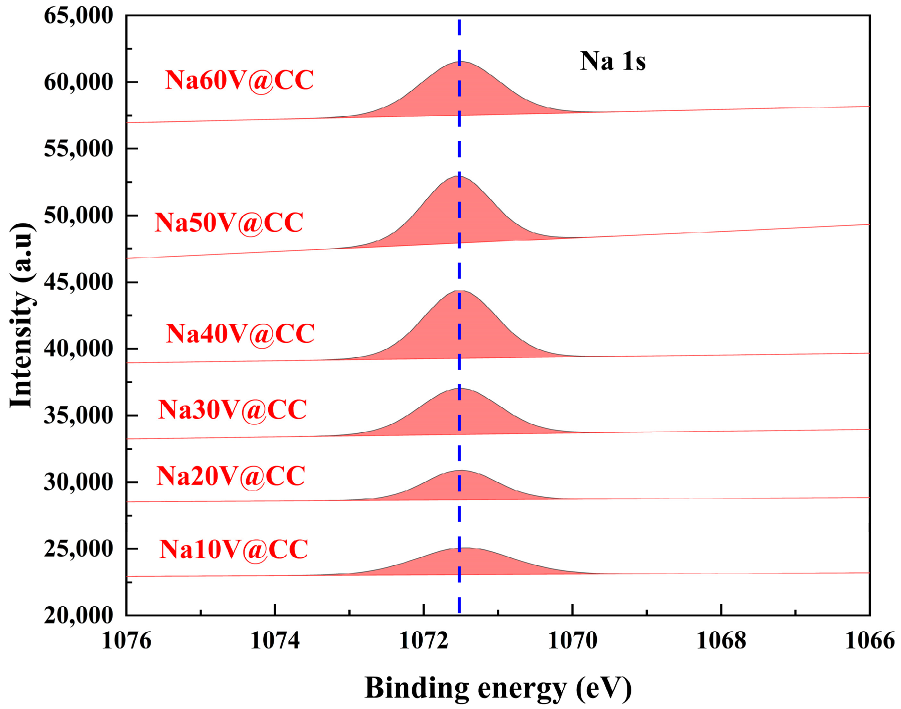

Fig.S14 The High-resolution XPS results of Na1s in Na10V@CC, Na20V@CC, Na30V@CC, Na40V@CC, Na50V@CC and Na60V@CC electrode **materials**

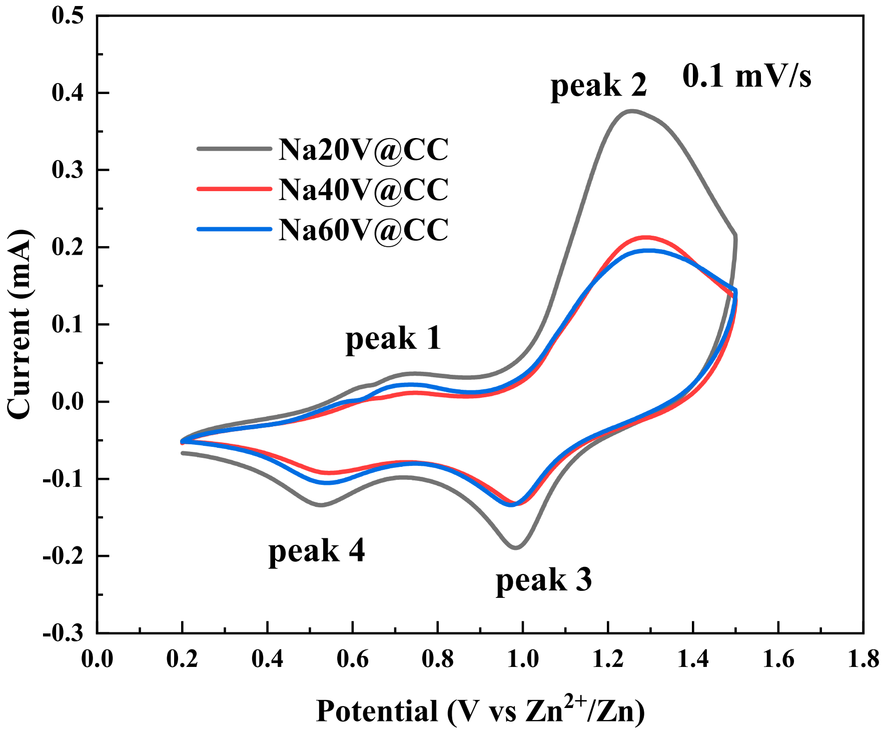

Fig.S15 The electrochemical CV scanning curves of Na20V@CC, Na40V@CC and Na60V@CC materials

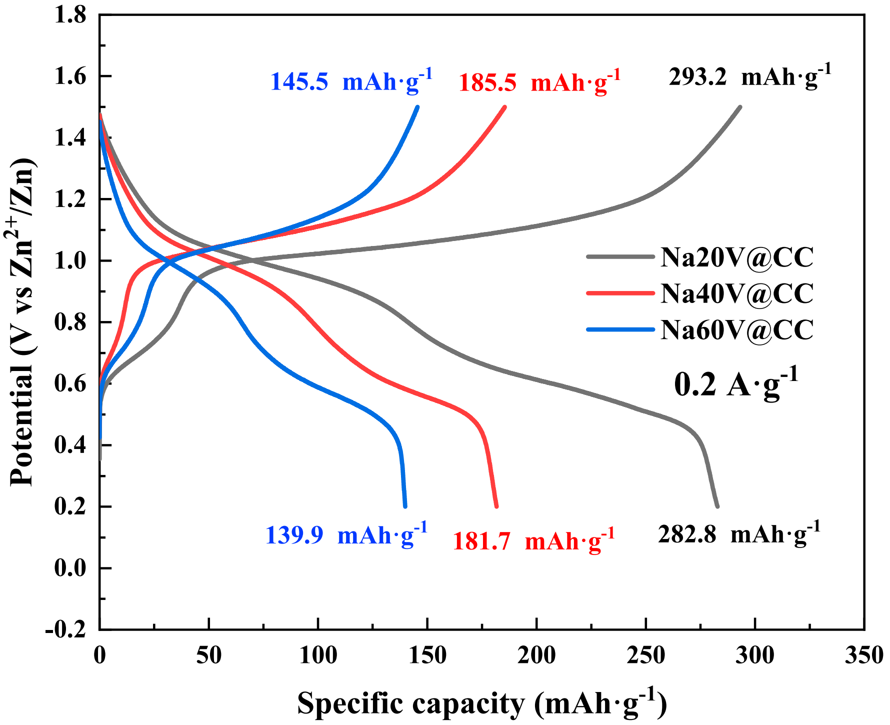

Fig.S16 The GCD curves (0.2 A·g-1) of Na20V@CC, Na40V@CC and Na60V@CC materials

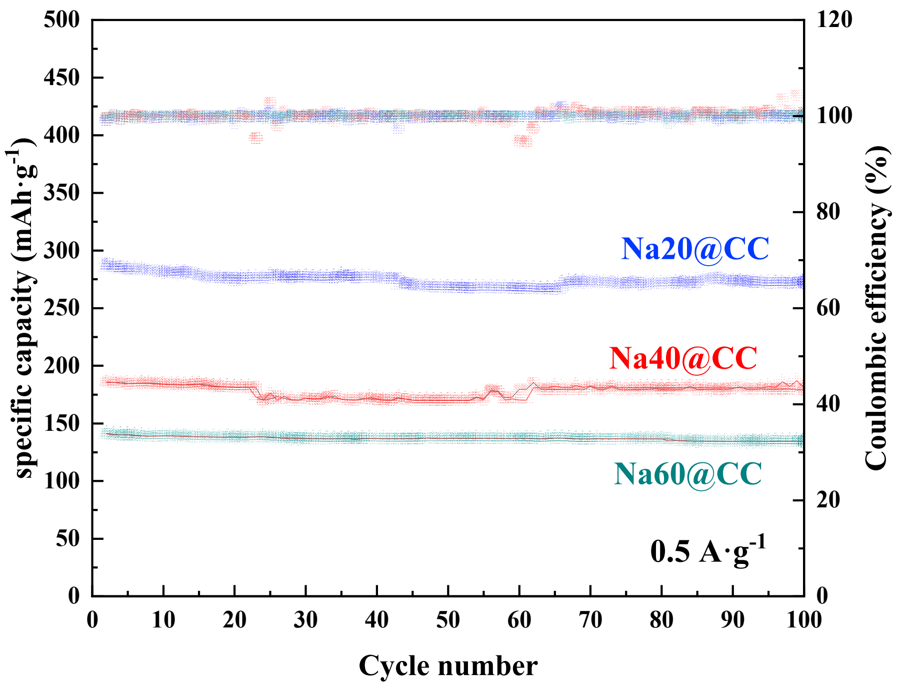

Fig.S17 The preliminary cyclic performance at 0.5 A·g^{-1} of Na20V@CC, Na40V@CC and Na60V@CC materials

Table.S1 The summary of recent electrochemical performance of cathode materials in ZIBs

Cathode Material	Potential Window (V)	Electrolyte	Capacity ($mAh\cdot g^{-1}$)	Cycle Performance (mAh/g)	References
$Mn_{0.2}V_8O_{20}\cdot 1.12H_2O$	0.2-1.6	2 M $Zn(CF_3SO_3)_2$	306.4 (0.1 $A\cdot g^{-1}$)	155.7 (after 1000 cycles at 2.0 $A\cdot g^{-1}$)	[1]
$K_{0.23}V_2O_5$	0.1-1.7	2 M $Zn(CF_3SO_3)_2$	284 (0.1 $A\cdot g^{-1}$)	103 (after 500 cycles at 2.0 $A\cdot g^{-1}$)	[2]
$Ag_2V_4O_{11}$-180	0.3–1.3	3 M $Zn(CF_3SO_3)_2$	251 (0.5 $A\cdot g^{-1}$)	117.6 (after 1000 cycles at 3.0 $A\cdot g^{-1}$)	[3]
$CaV_6O_{16}\cdot 3H_2O@Ni_{0.24}V_2O_5\cdot nH2O$	0.2-1.6	3 M $Zn(CF_3SO_3)_2$	334.7 (0.1 $A\cdot g^{-1}$)	82.6 (after 500 cycles at 2.0 $A\cdot g^{-1}$)	[4]
$Mn(VO_3)_2/MnV_{12}O_{31}\cdot 10H_2O$	0.2-1.8	3 M $Zn(CF_3SO_3)_2$	323.6 (0.5 $A\cdot g^{-1}$)	68.9 (after 2000 cycles at 3.0 $A\cdot g^{-1}$)	[5]
VOH-PANI/CC	0.2-1.6	3 M $Zn(CF_3SO_3)_2$	290 (0.5 $A\cdot g^{-1}$)	102 (after 1700 cycles at 4.0 $A\cdot g^{-1}$)	[6]
EG/VO_2	0.2-1.0	1 M $ZnSO_4$	345 (0.1 $A\cdot g^{-1}$)	108.9 (after 2000 cycles at 2.0 $A\cdot g^{-1}$)	[7]
VOH-AB/CC	0.2-1.6	3 M $Zn(CF_3SO_3)_2$	280 (1 $A\cdot g^{-1}$)	182 (after 3000 cycles at 4.0 $A\cdot g^{-1}$)	[8]
KMVO NPs-200	0.2-1.9	3 M $Zn(CF_3SO_3)_2$	150 (0.1 $A\cdot g^{-1}$)	98 (after 1000 cycles at 1.0 $A\cdot g^{-1}$)	[9]
$VO(acac)_2$@rGO	0.1-1.8	3 M $Zn(CF_3SO_3)_2$	327.2 (0.1 $A\cdot g^{-1}$)	71.1 (after 2000 cycles at 2.0 $A\cdot g^{-1}$)	[10]
$Ag_{0.3}V_2O_5$	0.2-1.6	3 M $Zn(CF_3SO_3)_2$	340 (0.1 $A\cdot g^{-1}$)	135 (after 1600 cycles at 2.0 $A\cdot g^{-1}$)	[11]
$CC@NH_4V_4O_{10}$	0.2-1.6	1 M $ZnSO_4$	330 (0.25 $A\cdot g^{-1}$)	107.3 (after 600 cycles at 5.0 $A\cdot g^{-1}$)	[12]
$PANI/O\text{-}V_2CT_x$	0.2-1.6	2 M $ZnSO_4$	267.7 (0.2 $A\cdot g^{-1}$)	109.9 (after 2000 cycles at 5.0 $A\cdot g^{-1}$)	[13]
$Ba\text{–}V_6O_{13}$	0.2-1.4	1 M $ZnSO_4$	305 (0.1 $A\cdot g^{-1}$)	106 (after 2500 cycles at 5.0 $A\cdot g^{-1}$)	[14]
Na30V@CC	0.2-1.5	2M $ZnSO_4$	343.3 (0.2 $A\cdot g^{-1}$)	138.9 (after 3000 cycles at 5 $A\cdot g^{-1}$)	This Work

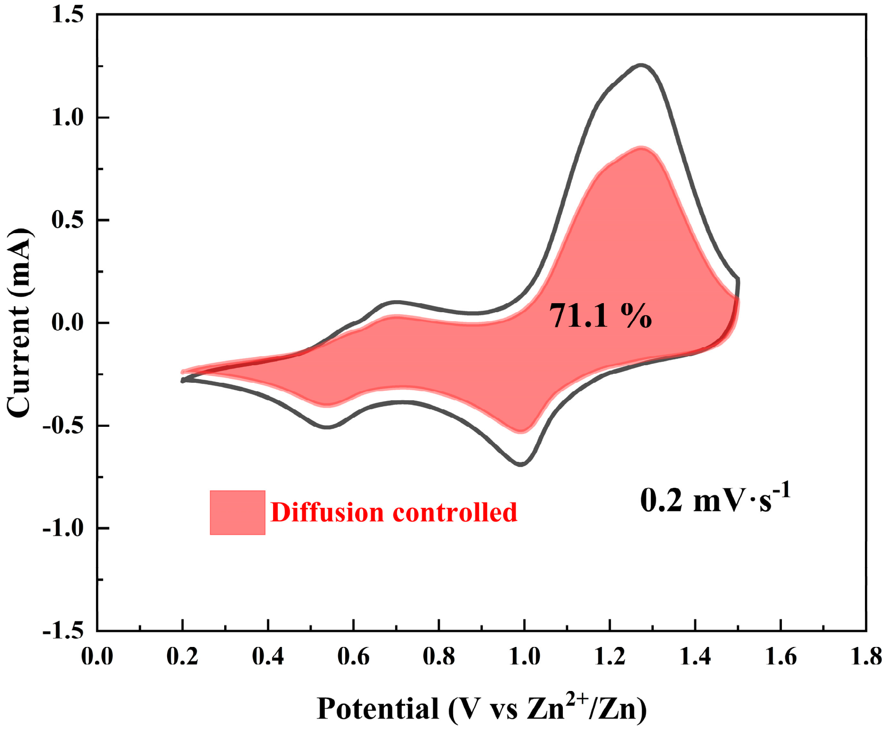

Fig.S18 The capacitive and diffusion contribution at 0.2 mV·s^{-1}

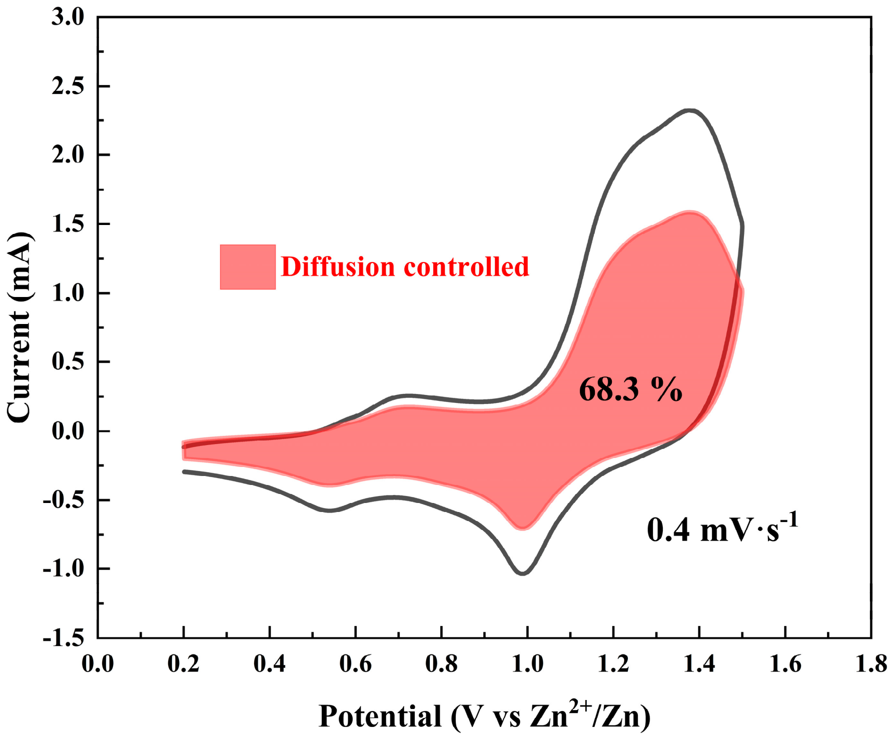

Fig.S19 The capacitive and diffusion contribution at 0.4 mV·s^{-1}

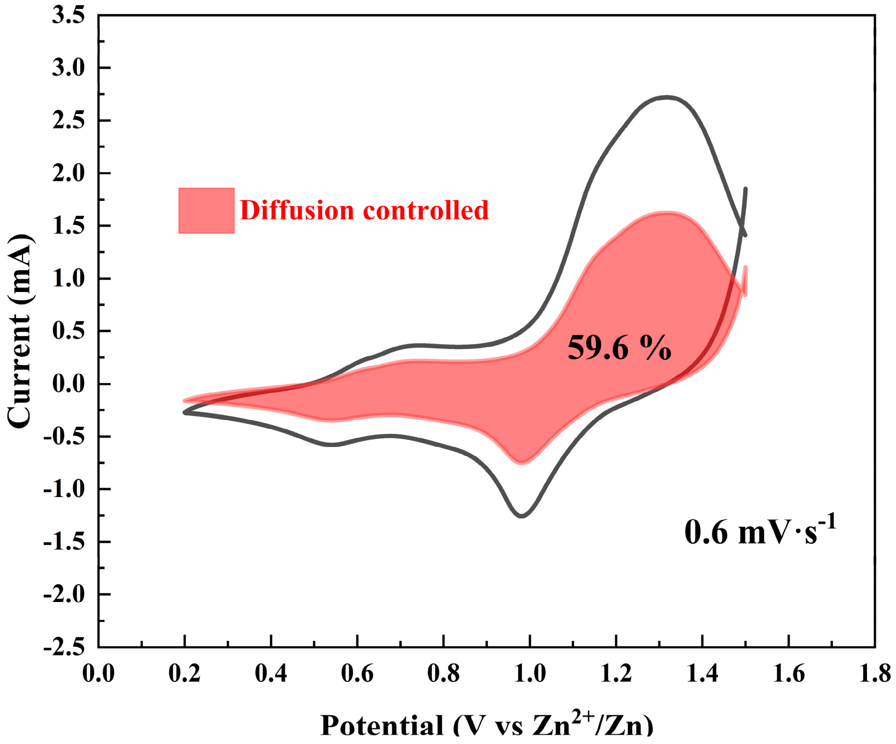

Fig.S20 The capacitive and diffusion contribution at 0.6 mV·s^{-1}

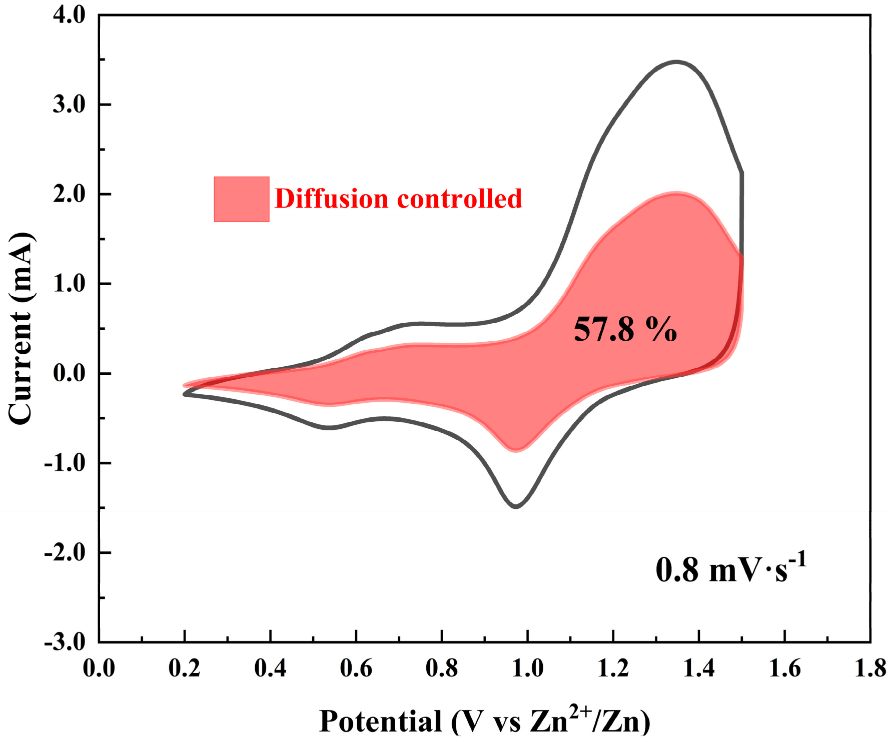

Fig.S21 The capacitive and diffusion contribution at 0.8 mV·s^{-1}

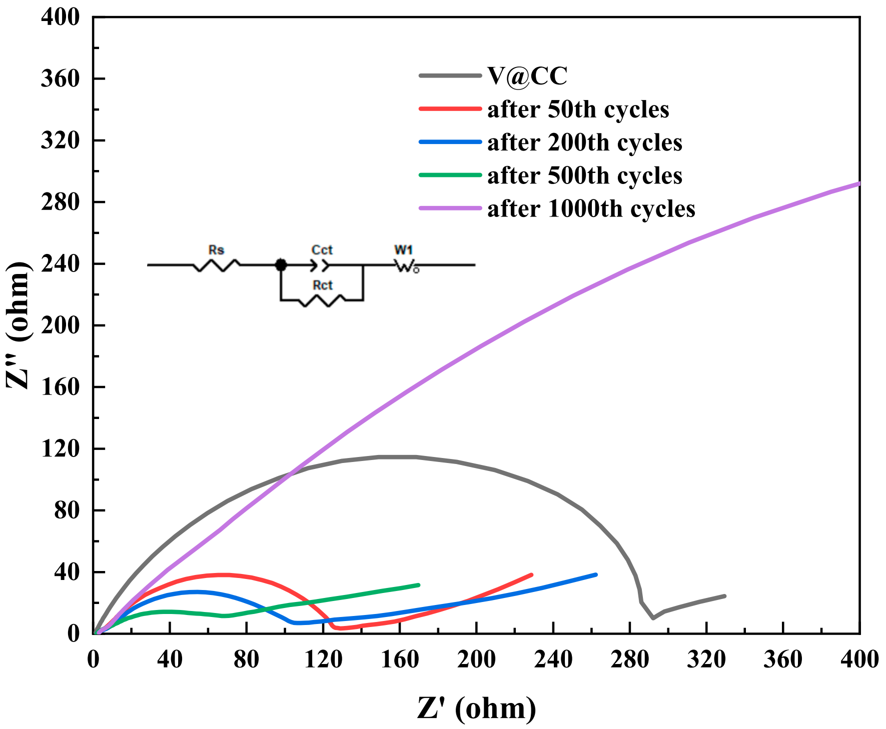

Fig.S22 The EIS curves and equivalent circuit model under different cycles for V@CC material (h: inserted picture is analog circuit)

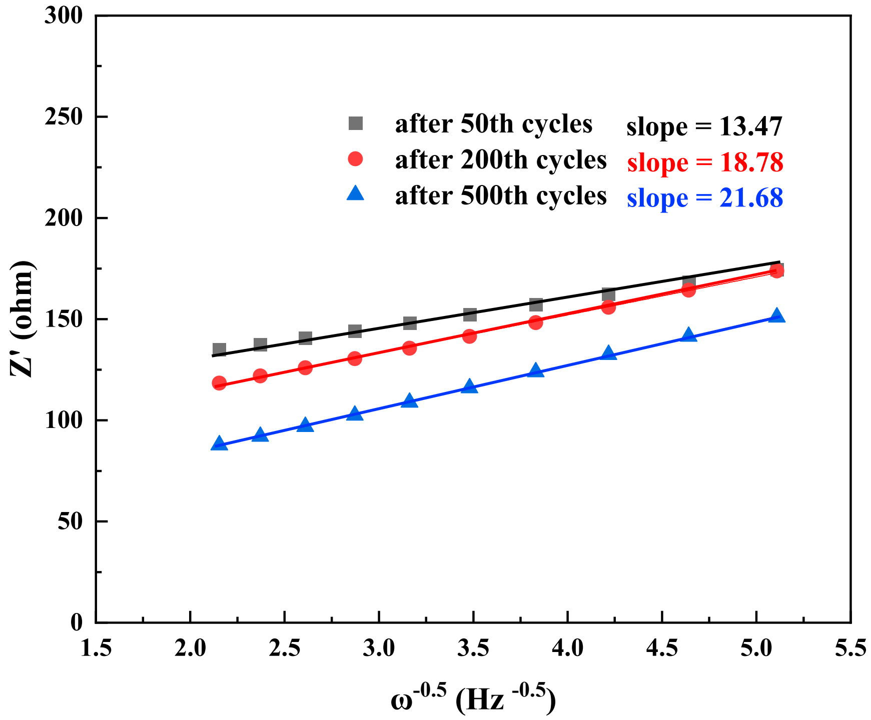

Fig.S23 The analysis results of linear relationship between Z' and $\omega^{-0.5}$ of V@CC material

Table.S2 The Zinc ion diffusion coefficient obtained from low-frequency range of EIS curves

V@CC			Na30V@CC		
ID	Zinc ion diffusion coefficient (D: $cm^2 \cdot s^{-1}$)	Zinc ion diffusion coefficient (log ($D\times10^{10}$))	ID	Zinc ion diffusion coefficient (D: $cm^2 \cdot s^{-1}$)	Zinc ion diffusion coefficient (log ($D\times10^{10}$))
			original	2.95×10^{-10}	0.469
after 50th cycles	6.94×10^{-10}	0.841	after 50th cycles	1.89×10^{-9}	1.276
after 200th cycles	3.57×10^{-10}	0.553	after 200th cycles	6.87×10^{-9}	1.837
after 500th cycles	2.68×10^{-10}	0.428	-		
			after 1000th cycles	1.004×10^{-8}	2.002
			after 2500th cycles	1.46×10^{-9}	1.165

The Zinc ion diffusion coefficient calculation formula [15,16]:

$$D = 0.5[\frac{Vm}{FA\sigma}(-\frac{dE}{dx})]^2 \qquad \text{(S1)}$$

Here, D is Zinc ion diffusion coefficient; σ is Warburg coefficient gained from analysis of low-frequency range of EIS curves (Fig.7i and S19); A is electrode area; Vm is molar volume of active substance; *dE/dx* is the slope of open circuit potential at a certain concentration on the zinc ion concentration curve in the electrode; F is Faraday constant.

The equations S1 was equivalent S2:

$$D = 0.5(\frac{RT}{n^2F^2AC\sigma})^2 \qquad \text{(S2)}$$

Here, R is gas constant; T is absolute temperature; n is the charge of zinc ions; C is the concentration of zinc ions in the electrode; The other parameters are the same as equation S1.

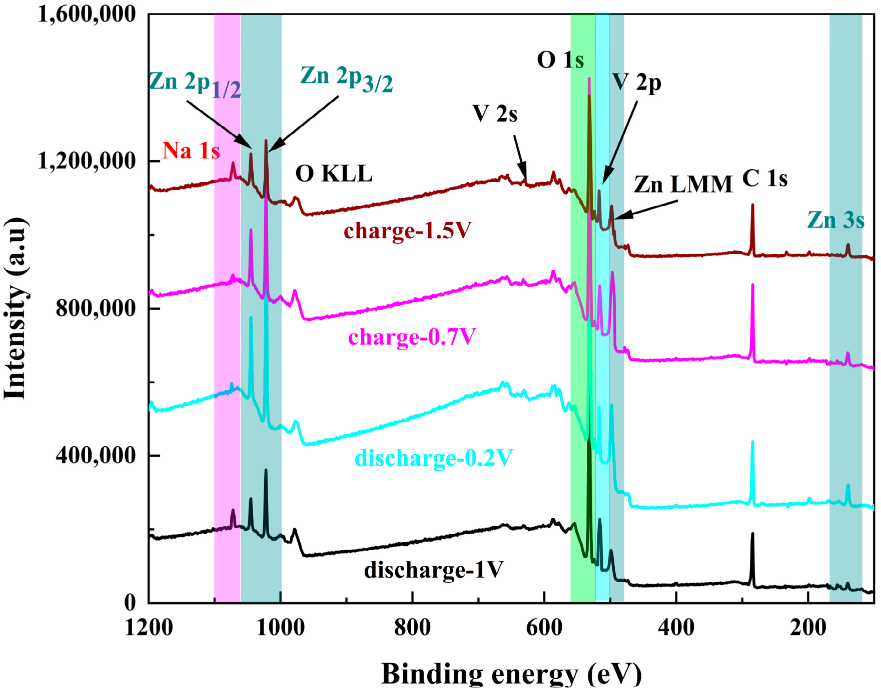

Fig.S24 The XPS full spectrum scanning result of charge-1.5V, charge-0.7V, charge-0.2V and charge-1V statuses

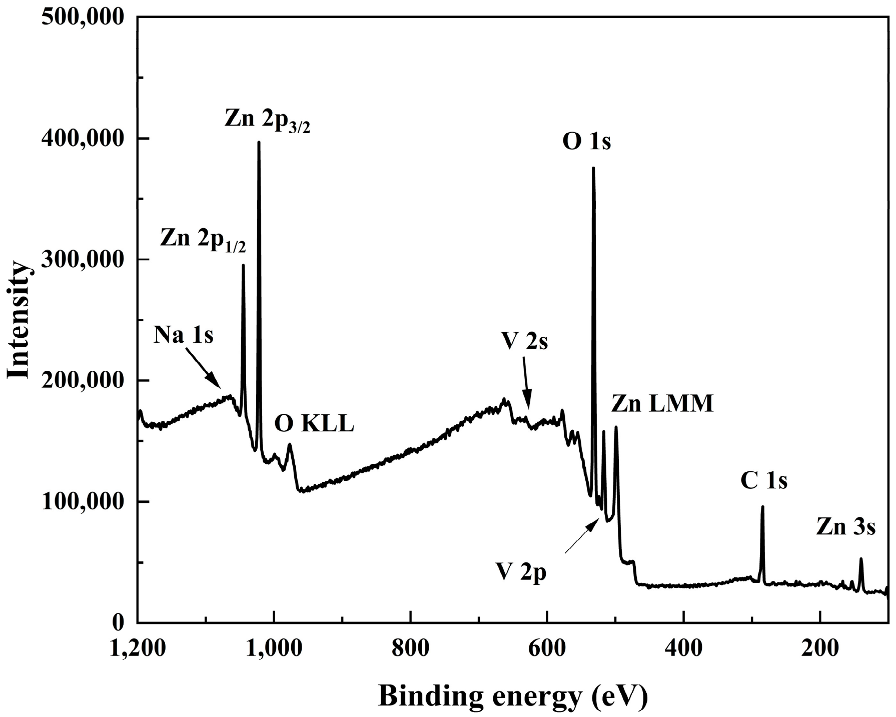

Fig.S25 The XPS full spectrum scanning result after 2500 charge-discharge cycles

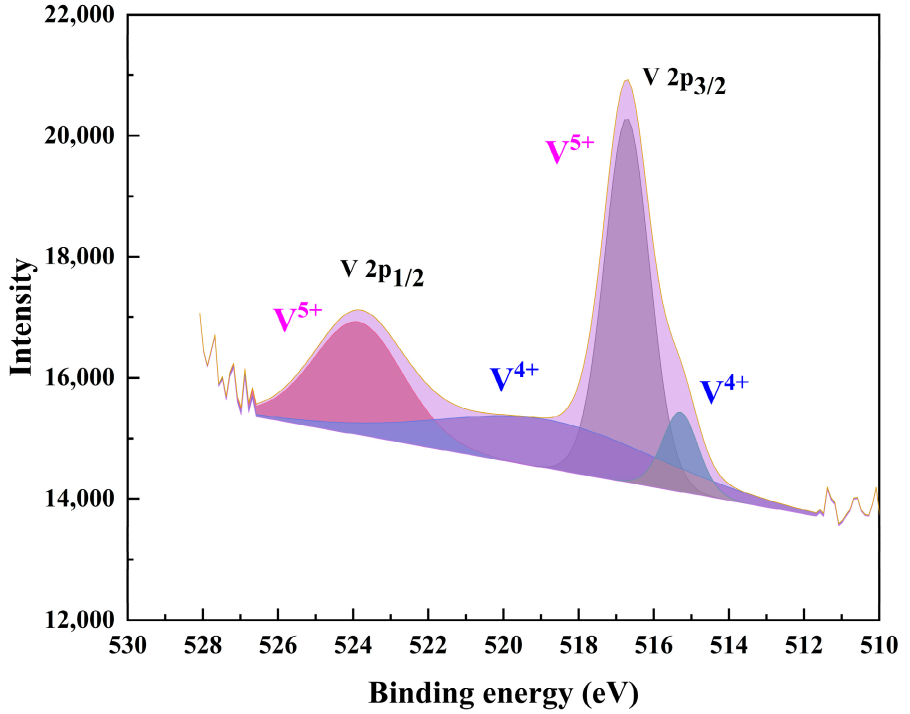

Fig.S26 The high-resolution V2p analysis result after 2500 charge-discharge cycles

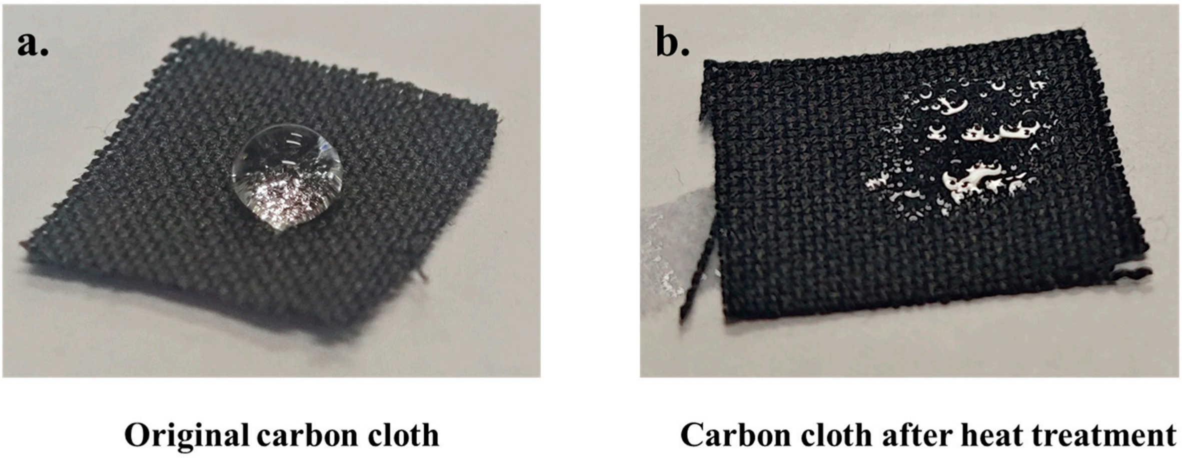

Fig.S27 The digital photos of hydrophilicity testing of original carbon cloth and carbon cloth after heat treatment

References

1. Pang, X.; Ji, S.; Zhang, P.; Feng, W.; Zhang, L.; Li, K.; Tang, Y.; Liu, Y. Interlayer doping of pseudocapacitive hydrated vanadium oxide via Mn2+ for high-performance aqueous zinc-ion battery. *Electrochimica Acta* **2023**, *441*, 141810. https://doi.org/10.1016/j.electacta.2022.141810.
2. Zhang, W.; Tang, C.; Lan, B.; Chen, L.; Tang, W.; Zuo, C.; Dong, S.; An, Q.; Luo, P. K0.23V2O5 as a promising cathode material for rechargeable aqueous zinc ion batteries with excellent performance. *Journal of Alloys and Compounds* **2020**, *819*, 152971. https://doi.org/https://doi.org/10.1016/j.jallcom.2019.152971.
3. Tong, X.; Zhong, J.; Hu, X.; Zhang, F. Preparation and Performance of Highly Stable Cathode Material Ag2V4O11 for Aqueous Zinc-Ion Battery. *Crystals* **2023**, *13*, 565. https://doi.org/10.3390/cryst13040565.
4. Zhang, S.; Wang, Y.; Wu, Y.; Zhang, G.; Chen, Y.; Wang, F.; Fan, L.; Yang, L.; Wu, Q. Hydrated Metal Vanadate Heterostructures as Cathode Materials for Stable Aqueous Zinc-Ion Batteries. *Molecules* **2024**, *29*, 3848. https://doi.org/doi.org/10.3390/molecules29163848.
5. Tan, Y.; Niu, X.; Chen, J. Phase regulation of manganese vanadium oxide and its effects on capacity for aqueous zinc-ion battery. *Journal of Energy Storage* **2024**, *99*, 113230. https://doi.org/10.1016/j.est.2024.113230.
6. Ye, S.; Sheng, S.; Wang, Y.; Li, J.; Li, Q.; Meng, L.; Chen, Q.; Yao, H. Synthesis of binder-free hydrated vanadium oxide-polyaniline electrodes via in situ polymerization for high-performance aqueous zinc-ion batteries. *Inorganic Chemistry Communications* **2024**, *166*, 112656. https://doi.org/10.1016/j.inoche.2024.112656.
7. Li, J.; Zhao, J.; Wang, Z.; Liu, H.; Wen, Q.; Yin, J.; Wang, G. Preparation of Expanded Graphite-VO2 Composite Cathode Material and Performance in Aqueous Zinc-Ion Batteries. *Materials (Basel)* **2024**, *17*, 2817. https://doi.org/doi.org/10.3390/ma17122817.
8. Ye, S.; Sheng, S.; Chen, Q.; Meng, L.; Yao, W.; Yao, H.; Wu, Z.; Zhang, F. Layer-by-layer assembled binder-free hydrated vanadium oxide-acetylene black electrode for flexible aqueous zinc ion battery. *Journal of Electroanalytical Chemistry* **2024**, *964*, 118334. https://doi.org/10.1016/j.jelechem.2024.118334.
9. Xiao, H.; Li, R.; Zhu, L.; Chen, X.; Xie, L.; Han, Q.; Qiu, X.; Yi, L.; Cao, X. Mn-containing heteropolyvanadate nanoparticles as a high-performance cathode material for aqueous zinc-ion batteries. *Journal of Energy Storage* **2024**, *89*, 111640. https://doi.org/10.1016/j.est.2024.111640.
10. Li, Y.; Jin, Y.; Zhou, H.; Fan, Q.; Dong, Y.; Kuang, Q.; Zhao, Y. Small organic molecules for aqueous zinc-ion batteries with stable structure and ultrafast H-ion and Zn-ion diffusion kinetics via coating. *Journal of Power Sources* **2024**, *603*, 234434. https://doi.org/10.1016/j.jpowsour.2024.234434.
11. Sun, Q.; Hu, L.; Cai, H.; Yi, Z.; Wang, L.; Liang, J.; Ni, J.; Zhang, J. Silver ion in combination intercalation/deintercalation reaction of aqueous zinc-ion batteries. *Journal of Materials Science* **2023**, *58*, 12008-12019. https://doi.org/10.1007/s10853-023-08757-0.
12. Nie, W.; Sun, P.; Xu, S.; Sun, J.; Sun, S.; Sun, Y.; Liu, L.; Xu, Z.; Jia, H. Self-supported carbon cloth based-ammonium vanadate nanoribbons cathode for superior flexible aqueous zinc-ion batteries. *Colloids and Surfaces A: Physicochemical and Engineering Aspects* **2023**, *674*, 131891. https://doi.org/10.1016/j.colsurfa.2023.131891.
13. Wang, L.; Lai, Y.; Tian, H.; Wang, J.; Zhao, W.; Wang, Y.; Li, L.; Zhang, L. Cooperative energy storage behaviors derived from PANI and V2CTx MXene for advanced aqueous zinc-ion batteries. *Journal of Alloys and Compounds* **2023**, *945*, 169366. https://doi.org/10.1016/j.jallcom.2023.169366.
14. Kulkarni, P.; Jung, H.Y. In-situ construction of barium-induced cathode electrolyte interphase to enable mechanostable high-performance zinc-ion batteries. *Materials Today Energy* **2023**, *32*, 101254. https://doi.org/10.1016/j.mtener.2023.101254.

15. Nguyen, T.Q.; Breitkopf, C. Determination of Diffusion Coefficients Using Impedance Spectroscopy Data. *Journal of The Electrochemical Society* **2018**, *165*, E826-E831. https://doi.org/10.1149/2.1151814jes.

16. SuXia, S.; Zhendong, Z.; Rongrong, W.; Wen, P. Characterizing diffusion coefficient of electrode materials by three methods. *Battery Bimonthly (in Chinese)* **2021**, *51*, 577-581. https://doi.org/10.19535/j.1001-1579.2021.06.008.
